# Geometric analysis of non-degenerate shifted-knots Bézier surfaces in Minkowski space

**DOI:** 10.1371/journal.pone.0296365

**Published:** 2024-01-03

**Authors:** Sadia Bashir, Daud Ahmad

**Affiliations:** Department of Mathematics, University of the Punjab, Lahore, Pakistan; Huazhong University of Science and Technology, CHINA

## Abstract

In this paper, we investigate the properties of timelike and spacelike shifted-knots Bézier surfaces in Minkowski space-$E_{1}^{3}$. These surfaces are commonly used in mathematical models for surface formation in computer science for computer-aided geometric design and computer graphics, as well as in other fields of mathematics. Our objective is to analyze the characteristics of timelike and spacelike shifted-knots Bézier surfaces in Minkowski space-$E_{1}^{3}$. To achieve this, we compute the fundamental coefficients of shifted-knots Bézier surfaces, including the Gauss-curvature, mean-curvature, and shape-operator of the surface. Furthermore, we present numerical examples of timelike and spacelike bi-quadratic (*m* = *n* = 2) and bi-cubic (*m* = *n* = 3) shifted-knots Bézier surfaces in Minkowski space-$E_{1}^{3}$ to demonstrate the applicability of the technique in Minkowski space.

## 1 Introduction

Differential geometry (DG) deals with the geometry of curves, surfaces, and manifolds, which are higher-dimensional analogues of surfaces. It has a long history dating back to the work of Gauss and Riemann, and has become one of the most active areas of research in mathematics in recent decades. This is partly due to its intersection with many other mathematical fields, including partial differential equations, metric and discrete geometry, topology, geometric and Lie group theory, and stochastics on manifolds. Furthermore, DG has many applications in fields such as theoretical physics, robotics, machine learning, computer vision, and engineering, and is used in cutting-edge research in areas such as robotically assisted nano-precision surgery, protein design, and manifold learning. It is also the mathematical foundation of Einstein’s theory of general relativity, and forms the basis of all modern positioning technologies, including the Global positioning system (GPS). The study of curves and surfaces in space can be traced back to the development of calculus in the 17th century, which made it possible to examine more complicated plane curves. The study of integral calculus in particular produced general solutions to ancient problems of arc length and area of plane figures. These solutions paved the way for the study of curves and surfaces in space, which marked the beginning of the field of differential geometry. Differential geometry provides a precise method for measuring the curvature of a curve or surface. The study of surface curvature at a particular point was first examined by Euler in 1760, who looked at cross sections of a surface created by planes that contain the normal line to the surface at that point. DG is the fundamental tool for understanding the properties and characteristics of shifted-knots Bézier surfaces in Minkowski space-$E_{1}^{3}$, a non-Euclidean three-dimensional space. By utilizing these concepts and techniques, one can compute key characteristics of these surfaces such as Gaussian curvature, mean curvature, and the shape operator. The shape operator of a Bézier surface is a linear operator that describes the local behavior of the surface in a given direction. It can be used to calculate the principal curvatures, normal vectors, and other geometric properties of the surface. In the case of a Bézier surface that has been shifted along a knot, the shape operator depends on the specific shift and the properties of the underlying Bézier surface and in Minkowski space-$E_{1}^{3}$, it also depends on the properties of the underlying space, its dimension and the presence of any curvature. The shape operator, also known as the Weingarten map, describes the local behavior of the surface at a specific point and can be used to calculate the principal curvatures and normal vectors of the surface. These characteristics provide valuable insights into the local behavior of the surface at a specific point, which have a wide range of applications including computer graphics, geometric modeling, and computer-aided design (CAD) systems.

In the 1960s, two French automobile engineers, Bézier and Casteljau, who worked for Renault and Citroen respectively, independently developed a method for effectively designing and manipulating smooth curves. These solutions were initially considered industrial secrets, but Bézier’s work was eventually published first. The curves that result from this method are now known as Bézier curves. Bézier curves are widely used in computer graphics and other computer drawing systems due to their ability to enable quick manipulation of shapes, including rotation, distortion, and stretching, while efficiently storing their information. They are also used to define the shapes of letters in various fonts, allowing for the creation of numerous typefaces in different sizes with minimal memory usage. Bézier tensor-product surfaces are the extension of Bézier curves to higher dimensions, which are widely used in engineering and computer graphics. In recent years, there has been an increasing focus on parallelization approaches to speed up the computation of Bézier surfaces, particularly in the field of graphics applications. Despite decades of research on Bézier curves and surfaces, there is still much to learn about them, and their use continues to be essential in the representation and communication of geometric data. In recent years, Bézier curves and surfaces in the Euclidean space-$E^{3}$ have been extensively studied, as demonstrated by various publications such as [1–12]. These studies have focused on different aspects of Bézier curves and surfaces, including their properties, applications, and computational methods. This includes the investigation of variational improvement in Bézier surfaces using quasi-minimal functions [13, 14], the applications of Bézier curves and surfaces in computer-aided geometric design and modeling [15–17]. Furthermore, Bézier surfaces have been employed to solve the Plateau-Bézier problem, which involves finding a Bézier surface of minimal area among all possible surfaces spanned by the same boundary [2, 18, 19]. The utilization of variational techniques, including the extremization of Dirichlet and energy functionals, to tackle the Plateau-Bézier problem has been significant [7, 20–23]. Additionally, numerical methods and energy functionals have been applied to obtain quasi-minimal surfaces [3, 5–7, 9, 24–27]. Recent studies on the Serret-Frenet frame, such as the work by Samanci et al. [28], have provided further insights into this topic. In the field of free field measurements under aided conditions, Razzaq et al. [29] introduced a new loudness function that incorporates Bézier interpolation to improve accuracy. Pradhan and Mohan [30] presented a novel method for maintaining the continuity of contour lines on topographic sheets, integrating the principles of Sign of Gradient (SG), Euclidean Distance (ED), and a customized Bézier Curve (BC) drawing method. Cao et al. [31] proposed an efficient multi-degree reduction method for Ball Bézier surfaces under varying interpolation constraints, employing metaheuristic methods.

The study of geometric objects from mathematical and computational perspectives is the main concern of computational geometry. In this field, the construction of curves and surfaces often involves the use of Bernstein operators and their generalizations. Bézier curves are a well-known example in computational geometry that are constructed using Bernstein bases. Recently, Khalid et.al. [32] demonstrated the use of shifted-knot Bernstein operators on the modified Bernstein bases to create Bézier curves and surfaces. Bernstein polynomials are also widely used in well-known software such as Adobe Illustrator and Flash [33], as well as font imaging systems like PostScript [34]. The Stancu-type generalization [35] of the family of Bernstein-Kantorovich operators [36], which involves the parameter *β* ∈ [0, 1] with shifted-knots, has also been introduced by Pratap and Deo [37]. Mursaleen and Qasim [38] proposed this type of generalization for the Stancu-type polynomials for the *q*-analog of Lupas-Bernstein-operators with shifted-knots. For the purpose of constructing *q*-Bézier curves and surfaces, Kottakkaran, Vinita and Asif [39] developed blending functions of Lupas *q*-Bernstein operators with shifted-knots. Gadjiev and Gorhanalizadeh [40] introduced the construction of Bernstein Stancu-type polynomials with shifted-knots. Mursaleen, Kilicman and Nasiruzzaman [41] designed the shifted-knots of Bernstein-Kantorovich operators generated by the basic *q*-calculus. Shagufta, Mursaleen and Maria [42] introduced Kantorovich variant of *λ*-Bernstein operators with shifted-knots.

Minkowski addressed the problems in relativity theory by the use of the geometry, now known as Minkowski geometry. He generalized Riemannian-metric problems into Lorentz-Minkowski spaces using a pseudo-Riemannian metric. In Minkowski space-$E_{1}^{3}$, vectors are divided into timelike, lightlike, and spacelike by the Lorentz-Minkowski metric. These vectors have a causal nature, which can make seemingly straightforward problems complicated, particularly those involving null-vectors, such as pseudo-null-curves, null-curves, marginally trapped surfaces, and *B*-scrolls. One issue is the inability to accurately measure angles related to lightlike-vectors, which limits some studies. On the non-degenerate surfaces, timelike surfaces, and spacelike surfaces in Minkowski space-$E_{1}^{3}$, numerous investigations have been done. For example, Treibergs [43] has investigated spacelike hypersurfaces of constant mean-curvature in the Minkowski space-$E_{1}^{3}$. For timelike-surfaces with a defined Gauss-map, Aledo et al. [44] get a Lelievvre-type representation. In the Minkowski space-$E_{1}^{3}$, Abdel-Baky and Abd-Ellah [45] investigate both (spacelike and timelike) governed *W*-surfaces. Brander et al. [46] used the non-compact real form *SU* to construct spacelike constant mean-curvature surfaces in the Minkowski space-$E_{1}^{3}$. Lin [47] studied the impacts of curvature restrictions on timelike-surfaces in the Minkowski space-$E_{1}^{3}$ that are convex in the same way as are the surfaces in the Euclidean space-$E_{1}^{3}$. Kossowski [48] explored zero mean-curvature surface constraints in the Euclidean space-$E_{1}^{3}$. In his work, Georgiev [49] found sufficient conditions for Bézier surfaces to be spacelike. Ugail et al. [50] analyzed Bézier surfaces in the three-dimensional Minkowski space-$E_{1}^{3}$, considering both timelike and spacelike cases, and sought to determine the surfaces that are extremals of the Dirichlet functional. Kuşak Samancı and Celik [51] presented a geometric viewpoint of Bézier surfaces in Minkowski space and determined the shape operator of both timelike and spacelike Bézier surfaces in $E_{1}^{3}$. In this work, we find the fundamental coefficients, Gauss-curvature, mean-curvature, and shape-operator of the timelike and spacelike shifted-knots Bézier surface. The results are then used to illustrate the scheme for the associated shape-operator of the timelike and spacelike bi-quadratic and bi-cubic SKBS in the Minkowski space-$E_{1}^{3}$.

The paper is organized as follows: Section (2) covers the fundamental notation and definitions, as well as the conditions of timelike and spacelike shifted-knots Bézier surface in the Minkowski space-$E_{1}^{3}$. Section (3) is devoted to the related discussion and the results for the shape operator investigation of the timelike and spacelike SKBS, the Gauss-curvature and the mean-curvature. We include explanatory numeric examples in the section (4), for timelike and spacelike, bi-quadratic (*m* = *n* = 2) and bi-cubic (*m* = *n* = 3) SKBS in the Minkowski space-$E_{1}^{3}$ as an application of the technique developed in the section (3). Finally, section (5) contains the final remarks and the future prospects of the work.

## 2 Preliminaries

In this section, we provide an overview of the key concepts that will be used in the subsequent sections of our work. The three dimensional Euclidean and Minkowski spaces are denoted by $E^{3}$ and $E_{1}^{3}$, respectively. This includes the classical form of Bernstein polynomials, Bézier surfaces in classical Bernstein polynomials [52, 53], and shifted-knots Bernstein polynomials with the corresponding Bézier surface in Euclidean space-$E^{3}$ [39, 54], the inner-product and the cross-product of two vectors in Minkowski space-$E_{1}^{3}$ for the fundamental coefficients, shape operator, mean curvature, Gaussian curvature [55], and the derivatives of the respective shifted-knots Bézier surfaces. A Bézier curve in Euclidean space-$E^{3}$, denoted by L(u), is defined by a set of (*n* + 1) control points, $P_{0}$, $P_{1}$, …, $P_{n}$, and a set of basis functions, $B_{\ell }^{n}\left(u\right)$, which are the *n*^*th*^ degree Bernstein polynomials, as given by the following equation,

$$\begin{array}{c} L\left(u\right)=\sum_{\ell =0}^{n}B_{\ell }^{n}\left(u\right)P_{\ell }. \end{array}$$ (2.1)

The *n*^*th*^ degree Bernstein polynomials $B_{\ell }^{n}\left(u\right)$ are defined as

$$\begin{array}{c} B_{\ell }^{n}\left(u\right)=C_{\ell }^{n}\;u^{\ell }{\left(1-u\right)}^{n-\ell }, \end{array}$$ (2.2)

where Cℓn=(nℓ), for 0 ≤ *n* ≤ *ℓ*. A Bézier surface L(u,v) in Euclidean space-$E^{3}$ is defined by a set of control points, $P_{00}$, $P_{01}$,…, $P_{mn}$ and a set of basis functions, $B_{k}^{m}\left(u\right)$ and $B_{l}^{n}\left(v\right)$ as indicated in the following equation,

$$\begin{array}{c} L\left(u,v\right)=\sum_{k,l=0}^{m,n}B_{k}^{m}\left(u\right)B_{l}^{n}\left(v\right)P_{kl}, \end{array}$$ (2.3)

for 0 ≤ *m* ≤ *k*, 0 ≤ *n* ≤ *l* and (*u*, *v*) ∈ [0, 1] × [0, 1]. A shifted-knots Bézier curve (SKBC), ***ω***(*v*), in Euclidean space-$E^{3}$ is defined by a set of (*n* + 1) control points, $P_{0}$, $P_{1}$,…, $P_{n}$ and a set of basis functions, $S_{♭,\varsigma }^{l,n}\left(v\right)$,

$$\begin{array}{c} \omega \left(v\right)=\sum_{l=0}^{n}S_{♭,\varsigma }^{l,n}\left(v\right)\;P_{l}, \end{array}$$ (2.4)

for $v\in [\frac{♭}{n+\varsigma },\frac{n+♭}{n+\varsigma }]$, where $S_{♭,\varsigma }^{l,n}\left(v\right)$ are the *n*^*th*^ degree shifted-knots Bernstein polynomials. The *n*^*th*^ degree shifted-knots Bernstein polynomials $S_{♭,\varsigma }^{l,n}\left(v\right)$ are defined as

$$\begin{array}{c} S_{♭,\varsigma }^{l,n}\left(v\right)=\left(\begin{array}{c} n\\ l \end{array}\right){\left(\frac{n+\varsigma }{n}\right)}^{n}{\left(v-\frac{♭}{n+\varsigma }\right)}^{l}{\left(\frac{n+♭}{n+\varsigma }-v\right)}^{n-l}. \end{array}$$ (2.5)

**Fig 1:**
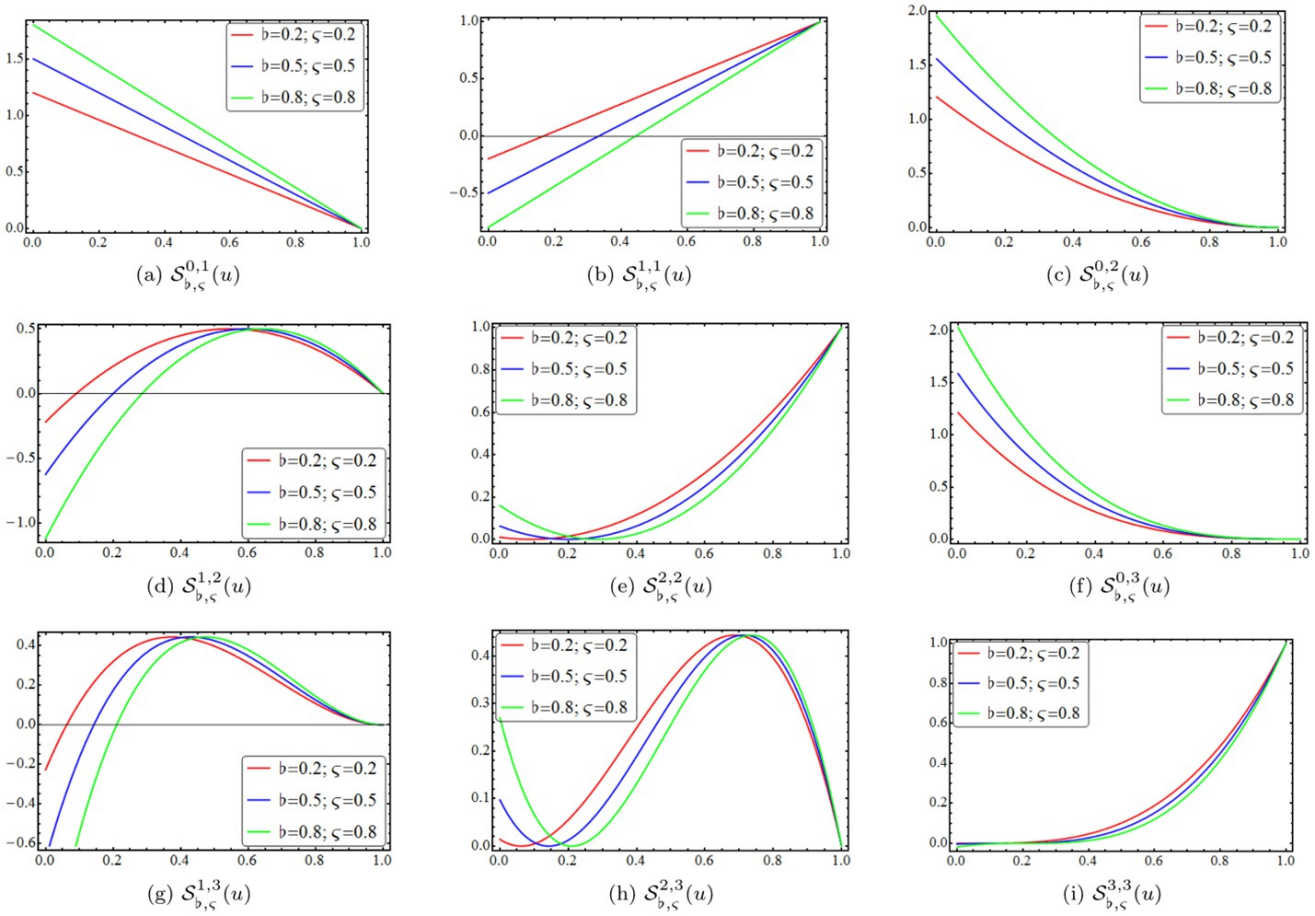
Shifted-knots Bernstein polynomials. The shifted-knots Bernstein polynomials $S_{♭,\varsigma }^{l,n}\left(v\right)$ of degree *n* = 1, 2, 3 for different values of ♭ and ς.

The *n*^*th*^-degree shifted-knots Bernstein polynomials $S_{♭,\varsigma }^{l,n}\left(v\right)$, given by the equations in (2.5), represent a family of polynomials used for polynomial approximation and interpolation. Each polynomial is characterized by its degree (*n*) and the parameters (♭ and ς), which control the shape and position of the polynomial curve. The expressions for the shifted-knots Bernstein polynomials $S_{♭,\varsigma }^{l,n}\left(v\right)$ given by above Eq (2.5) for *n* = 1, 2, 3 are as follows:

$$\begin{array}{cc} S_{♭,\varsigma }^{0,1}\left(v\right) & =(1+\varsigma )(\frac{1+♭}{1+\varsigma }-v),\\ S_{♭,\varsigma }^{1,1}\left(v\right) & =\left(\varsigma +1\right)(v-\frac{♭}{\varsigma +1}),\\ S_{♭,\varsigma }^{0,2}\left(v\right) & =\frac{1}{4}{\left(\varsigma +2\right)}^{2}{\left(\frac{♭+2}{\varsigma +2}-v\right)}^{2},\\ S_{♭,\varsigma }^{1,2}\left(v\right) & =\frac{1}{2}{\left(\varsigma +2\right)}^{2}(v-\frac{♭}{\varsigma +2})(\frac{♭+2}{\varsigma +2}-v),\\ S_{♭,\varsigma }^{2,2}\left(v\right) & =\frac{1}{4}{\left(\varsigma +2\right)}^{2}{\left(v-\frac{♭}{\varsigma +2}\right)}^{2},\\ S_{♭,\varsigma }^{0,3}\left(v\right) & =\frac{1}{27}{\left(\varsigma +3\right)}^{3}{\left(\frac{♭+3}{\varsigma +3}-v\right)}^{3},\\ S_{♭,\varsigma }^{1,3}\left(v\right) & =\frac{1}{9}{\left(\varsigma +3\right)}^{3}(v-\frac{♭}{\varsigma +3}){\left(\frac{♭+3}{\varsigma +3}-v\right)}^{2},\\ S_{♭,\varsigma }^{2,3}\left(v\right) & =\frac{1}{9}{\left(\varsigma +3\right)}^{3}{\left(v-\frac{♭}{\varsigma +3}\right)}^{2}(\frac{♭+3}{\varsigma +3}-v),\\ S_{♭,\varsigma }^{3,3}\left(v\right) & =\frac{1}{27}{\left(\varsigma +3\right)}^{3}{\left(v-\frac{♭}{\varsigma +3}\right)}^{3}, \end{array}$$

and they are shown in Fig 1 for varying values of ♭ and ς. Fig 1 depict the graphs of $S_{♭,\varsigma }^{0,1}(v)$, $S_{♭,\varsigma }^{1,1}(v)$, $S_{♭,\varsigma }^{0,2}(v)$, $S_{♭,\varsigma }^{1,2}(v)$, $S_{♭,\varsigma }^{2,2}(v)$, $S_{♭,\varsigma }^{0,3}(v)$, $S_{♭,\varsigma }^{1,3}(v)$, $S_{♭,\varsigma }^{2,3}(v)$ and $S_{♭,\varsigma }^{3,3}(v)$, which correspond to the shifted-knots Bernstein polynomials $S_{♭,\varsigma }^{l,n}\left(v\right)$ for *n* = 1, 2, 3 given by Eq (2.5). The specific values of ♭ and ς are ♭=0.2,0.5,0.8 and ς=0.2,0.5,0.8. For example, for l=0,n=1,♭=0.2,ς=0.2, we have: $S_{♭,\varsigma }^{0,1}(v)=1.2(1-v)$ and $S_{♭,\varsigma }^{1,1}(v)=1.2(v-0.166667)$. The Fig 1 illustrates the varying shapes and characteristics of the shifted-knots Bernstein polynomials $S_{♭,\varsigma }^{l,n}\left(v\right)$ as the parameters ♭ and ς change. A shifted-knots Bézier surface, ***ω***(*u*, *v*), is defined by a set of control points $P_{00}$, $P_{01}$,…, $P_{mn}$ in Euclidean space-$E^{3}$ as

$$\begin{array}{c} \omega \left(u,v\right)=\sum_{k=0}^{m}\sum_{l=0}^{n}S_{♭,\varsigma }^{k,m}(u)S_{♭,\varsigma }^{l,n}(v)P_{kl}. \end{array}$$ (2.6)

The shifted-knots Bézier curves on shifted-knots Bézier surfaces can be determined by keeping one of the parameters constant. These coordinate curves are known as *u*-parameter or the *v*-parameter curves and are expressed as ***ω***(*u*, *v*_0_) or ***ω***(*u*_0_, *v*). The coordinate curves ***ω***(*u*, 0), ***ω***(*u*, 1), ***ω***(0, *v*) and ***ω***(1, *v*) are the SKBS (compare it with Eq (2.4)). The coordinate curves ***ω***(*u*, 0), ***ω***(*u*, 1), ***ω***(0, *v*) and ***ω***(1, *v*) comprise the four edges of SKBS along with the endpoint interpolation at the corner-points

$$\begin{array}{c} \omega \left(0,0\right)=P_{00},\;\omega \left(1,0\right)=P_{m0},\;\omega \left(0,1\right)=P_{0m}\;and\;\omega \left(1,1\right)=P_{mn}, \end{array}$$ (2.7)

The parametric curves of the SKBS are shifted-knots Bézier curves (SKBCs) specifically, ***ω***(*u*, 0), ***ω***(*u*, 1), ***ω***(0, *v*) and ***ω***(1, *v*) are SKBCs that form the edges of the SKBS. The shifted knot Bézier surface (SKBS) remains unchanged under three-dimensional affine transformations, which preserve lines and parallelism. This is evident from the equation that for an affine transformation, T,

$$\begin{array}{c} T(\sum_{kl=0}^{m,n}S_{♭,\varsigma }^{k,m}(u)S_{♭,\varsigma }^{l,n}(v)P_{kl})=\sum_{kl=0}^{m,n}S_{♭,\varsigma }^{k,m}(u)S_{♭,\varsigma }^{l,n}(v)T(P_{kl}). \end{array}$$ (2.8)

This means that when an affine transformation T is applied to the SKBS, the resulting surface is the surface with the transformed control points. On the other hand, the Lorentz-Minkowski metric is defined by

$$\begin{array}{c} \left(\;,\;\right)={\left(ds^{1}\right)}^{2}+{\left(ds^{2}\right)}^{2}-{\left(ds^{3}\right)}^{2}, \end{array}$$ (2.9)

where (*s*^1^, *s*^2^, *s*^3^) are the canonical coordinates in the Euclidean space-$E^{3}$. For the two vectors ***μ*** = (*μ*^1^, *μ*^2^, *μ*^3^) and ***ν*** = (*ν*^1^, *ν*^2^, *ν*^3^), following are the definitions of the Lorentzian inner product and Lorentzian cross product,

$$\begin{array}{c} \xi_{L}(\mu ,\nu \;)=\mu^{1}\nu^{1}+\mu^{2}\nu^{2}-\mu^{3}\nu^{3}, \end{array}$$ (2.10)

and

$$\begin{array}{c} \mu \;\wedge_{L}\;\nu =-|\begin{array}{ccc} e^{1} & e^{2} & -e^{3}\\ \mu^{1} & \mu^{2} & \;\;\mu^{3}\\ \nu^{1} & \nu^{2} & \;\;\nu^{3} \end{array}|=(\mu^{3}\nu^{2}-\mu^{2}\nu^{3})e^{1}+(\mu^{1}\nu^{2}-\mu^{2}\nu^{1})e^{2}+(\mu^{1}\nu^{3}-\mu^{3}\nu^{1})e^{3}. \end{array}$$ (2.11)

When the vector ***μ*** is obtained in the Minkowski space-$E_{1}^{3}$, it is referred to as a spacelike-vector if *ξ*_*L*_(***μ***, ***μ***)>0 or ***μ*** = 0, it is lightllike-vector if *ξ*_*L*_(***μ***, ***μ***) = 0 or ***μ*** ≠ 0, and it is timelike-vector if *ξ*_*L*_(***μ***, ***μ***) < 0. The timelike and spacelike are the non-degenerate vectors. Assuming that *M* is a surface in the Minkowski space-$E_{1}^{3}$ with the parametrization ***ω*** = ***ω***(*u*, *v*) defined by $\omega :U\subset E^{2}\to E_{1}^{3}$. A tangent-plane $T_{P}M$ is said to pass through a point *P* of a regular plane *M* of the classes $C^{k}$, where *k* ≥ 1, when it is spanned by the vectors $\omega_{u}(P)$ and $\omega_{v}(P)$. A unit-normal **N** of a surface ***ω*** is defined by

$$\begin{array}{c} N={\left(\|\omega_{u}\wedge_{L}\omega_{v}\|\right)}^{-1}\left(\omega_{u}\;\wedge_{L}\omega_{v}\right)={\left(-\epsilon \left(EG-F^{2}\right)\right)}^{-\frac{1}{2}}\left(\omega_{u}\;\wedge_{L}\omega_{v}\right). \end{array}$$ (2.12)

The first-fundamental form on the plane $T_{P}M$ at the point P is represented by matrix,

$$\begin{array}{c} \eta =[\begin{array}{cc} E & F\\ F & G \end{array}], \end{array}$$ (2.13)

and has determinant det(*η*) = *EG* − *F*^2^. The coefficients *E*, *F* and *G* of the tangent plane $T_{P}M$ of the surface ***ω***(*u*, *v*) are defined by

$$\begin{array}{c} E=\xi_{L}(\omega_{u},\omega_{u}),\;F=\xi_{L}(\omega_{u},\omega_{v}),\;G=\xi_{L}(\omega_{v},\omega_{v}). \end{array}$$ (2.14)

The sign of the normal vector of a surface in Minkowski space-$E_{1}^{3}$ is determined by whether the surface is spacelike or timelike. For non-degenerate surfaces, the value *ξ*_*L*_(**N**, **N**) = *ϵ* serves as the determining factor to decide that if the surface is timelike or spacelike. If the surface is spacelike, the normal **N** is a time like vector, resulting in *ξ*_*L*_(**N**, **N**) = *ϵ* = −1, for the spacelike tangent plane. If the surface is timelike-surface, the normal **N** is a spacelike-vector since the tangent-plane is timelike, resulting in *ξ*_*L*_(**N**, **N**) = *ϵ* = 1. The norm of ***ω***_*u*_ ∧_*L*_ ***ω***_*v*_ is defined by

$$\begin{array}{c} \left\|\omega_{u}\wedge_{L}\omega_{v}\right\|={\left(-\epsilon \left(EG-F^{2}\right)\right)}^{\frac{1}{2}}={\left(-\epsilon \;\text{det}\left(\eta \right)\right)}^{\frac{1}{2}}. \end{array}$$ (2.15)

The coefficients of second-fundamental form *e*, *f* and *g* of the surface ***ω*** by using the Lorentzian-inner product (Eq (2.10)) are

$$\begin{array}{c} e=\xi_{L}(N,\omega_{uu}),\;f=\xi_{L}(N,\omega_{uv}),\;g=\xi_{L}(N,\omega_{vv}). \end{array}$$ (2.16)

Furthermore, using the first fundamental coefficients *E*, *F*, *G* (defined by Eq (2.14)) and the second fundamental coefficients *e*, *f*, *g* (defined by Eq (2.16)), the shape-operator of the surface ***ω***(*u*, *v*) is defined by the matrix

$$\begin{array}{c} \lambda =[\begin{array}{cc} \lambda_{11} & \lambda_{12}\\ \lambda_{21} & \lambda_{22} \end{array}]={\left[\begin{array}{cc} E & F\\ F & G \end{array}\right]}^{-1}[\begin{array}{cc} e & f\\ f & g \end{array}], \end{array}$$ (2.17)

where λ_11_, λ_12_, λ_21_ and λ_22_ are (from the above Eq (2.17)),

$$\begin{array}{c} \lambda_{11}=\frac{eG-fF}{\text{det}\;(\eta )},\;\lambda_{12}=\frac{fG-gF}{\text{det}\;(\eta )},\;\lambda_{21}=\frac{fE-eF}{\text{det}\;(\eta )},\;\lambda_{22}=\frac{gE-fF}{\text{det}\;(\eta )}. \end{array}$$ (2.18)

The mean-curvature *H* and the Gauss-curvature *K* for the non-degenerate surfaces (spacelike-surface or timelike-surface) can be obtained by corresponding shape-operator matrix $\lambda =[\begin{array}{cc} \lambda_{11} & \lambda_{12}\\ \lambda_{21} & \lambda_{22} \end{array}]$ of the surface,where,

$$\begin{array}{c} H=\frac{\epsilon \;tra(\lambda )}{2}=\frac{\epsilon (\lambda_{11}+\lambda_{22})}{2},\;K=\epsilon \;\text{det}\left(\lambda \right)=\epsilon (\lambda_{11}\;\lambda_{22}-\lambda_{12}\;\lambda_{21}). \end{array}$$ (2.19)

The first and second order partial derivatives of the shifted-knots Bernstein polynomials (2.5) are given by

$$\begin{array}{cc} {\left(S_{♭,\varsigma }^{l,n}\left(v\right)\right)}_{v} & =\left(n+\varsigma \right)\left(S_{♭,\varsigma }^{l-1,n-1}\left(v\right)-S_{♭,\varsigma }^{l,n-1}\left(v\right)\right)\\ {\left(S_{♭,\varsigma }^{l,n}\left(v\right)\right)}_{vv} & ={\left(n+\varsigma \right)}^{2}\left(\frac{n-1}{n}\right)\left(S_{♭,\varsigma }^{l-2,n-2}\left(v\right)-2S_{♭,\varsigma }^{l-1,n-2}\left(v\right)+S_{♭,\varsigma }^{l,n-2}\left(v\right)\right) \end{array}$$

leading to the following outcomes. These partial derivatives contribute to a reduction in the degree of the shifted-knot Bernstein polynomials (2.5). This reduction leads to a decrease in the degree of the Bernstein polynomials used in the corresponding shifted-knot Bézier curves (2.4) and (2.6). These results are presented for the sake of completeness and self-containment. For additional details, readers may refer to Ahmad et al.’s work [9].

**Theorem 2.1**. *The first order derivative* ***ω***_*v*_(*v*) *of SKBC, given in* (2.4), *in Euclidean space*-$E^{3}$ *defined for the control points* $P_{l}$ *is given by*

$$\begin{array}{c} \frac{d\omega }{dv}\left(v\right)=\sum_{\ell =0}^{n}{\left(S_{♭,\varsigma }^{l,n}\left(v\right)\right)}_{v}P_{l}=\sum_{l=0}^{n-1}S_{♭,\varsigma }^{l,n-1}(v)P_{l}^{\left(1\right)}, \end{array}$$ (2.20)

*where*

$$\begin{array}{c} P_{l}^{\left(1\right)}=(n+\varsigma )(P_{l+1}-P_{l}). \end{array}$$ (2.21)

**Theorem 2.2**. *The first order partial derivative* ***ω***_*u*_(*u*, *v*) *of SKBS, given in* (2.6), *with respect to the surface parameter* *u* *is*

$$\begin{array}{c} \omega_{u}\left(u,v\right)=\sum_{k=0}^{m-1}\sum_{l=0}^{n}S_{♭,\varsigma }^{k,m-1}(u)S_{♭,\varsigma }^{l,n}(v)P_{kl}^{\left(1,0\right)}, \end{array}$$ (2.22)

*where*,

$$\begin{array}{c} P_{kl}^{\left(1,0\right)}=(m+\varsigma )(P_{k+1,l}-P_{kl}), \end{array}$$ (2.23)

*is the weighted forward difference of control points* $P_{k+1,l}$ *and* $P_{kl}$. *In a similar manner, the surface parameter v can be used to calculate* ***ω***_*v*_(*u*, *v*) (*the first-order-partial derivative of shifted-knots Bézier surface*),

$$\begin{array}{c} \omega_{v}\left(u,v\right)=\sum_{k=0}^{m}\sum_{l=0}^{n-1}S_{♭,\varsigma }^{k,m}(u)S_{♭,\varsigma }^{l,n-1}(v)P_{kl}^{\left(0,1\right)}, \end{array}$$ (2.24)

*where*

$$\begin{array}{c} P_{kl}^{\left(0,1\right)}=(n+\varsigma )(P_{k,l+1}-P_{k,l}). \end{array}$$ (2.25)

**Corollary 2.2.1**. *The first-order partial derivatives of the SKBS with respect to the surface parameters u and v at the minimum-point* (*u*, *v*) = (0, 0) *can be obtained using* Eq (2.22) *through* (2.25) *and they are*

$$\begin{array}{c} \omega_{u}\left(0,0\right)=(m+\varsigma )(P_{10}-P_{00})=P_{00}^{\left(1,0\right)}, \end{array}$$ (2.26)

*and*

$$\begin{array}{c} \omega_{v}\left(0,0\right)=(n+\varsigma )(P_{01}-P_{00})=P_{00}^{\left(0,1\right)}. \end{array}$$ (2.27)

**Theorem 2.3**. *The second-order partial derivatives of SKBS with respect to the surface parameter u can be defined by using* Eq (2.22). *It is given by*,

$$\begin{array}{c} \omega_{uu}\left(u,v\right)=\sum_{k=0}^{m-2}\sum_{l=0}^{n}S_{♭,\varsigma }^{k,m-2}(u)S_{♭,\varsigma }^{l,n}(v)P_{kl}^{\left(2,0\right)}, \end{array}$$ (2.28)

*where*

$$\begin{array}{c} P_{kl}^{\left(2,0\right)}={\left(m+\varsigma \right)}^{2}(\frac{m-1}{m})(P_{k+2,l}-P_{k+1,l}+P_{k,l}). \end{array}$$ (2.29)

*The second-order-partial derivative of the SKBS w*.*r*.*t*. *the surface parameter v is defined by using the* Eq (2.22)

$$\begin{array}{c} \omega_{uv}\left(u,v\right)=\sum_{k=0}^{m-1}\sum_{l=0}^{n-1}S_{♭,\varsigma }^{k,m-1}(u)\;S_{♭,\varsigma }^{l,n-1}(v)P_{kl}^{\left(1,1\right)}, \end{array}$$ (2.30)

*where*,

$$\begin{array}{c} P_{kl}^{\left(1,1\right)}=(m+\varsigma )(n+\varsigma )(P_{k+1,l+1}-P_{k+1,l}-P_{k,l+1}+P_{k,l}). \end{array}$$ (2.31)

*In a similar fashion, the second-order partial derivative of shifted-knots Bézier surface with respect to the surface parameter v can be defined using* Eq (2.24), *given by*,

$$\begin{array}{c} \omega_{vv}\left(u,v\right)=\sum_{k=0}^{m}\sum_{l=0}^{n-2}S_{♭,\varsigma }^{k,m}(u)\;S_{♭,\varsigma }^{l,n-2}(v)P_{kl}^{\left(0,2\right)}, \end{array}$$ (2.32)

*where*,

$$\begin{array}{c} P_{kl}^{\left(0,2\right)}={\left(n+\varsigma \right)}^{2}\left(\frac{n-1}{n}\right)(P_{k,l+2}-P_{k,l+1}+P_{k,l}). \end{array}$$ (2.33)

**Corollary 2.3.1**. *The value of* ***ω***_*uu*_ (0, 0) *of the second order partial derivatives of SKBS* *w.r.t* *the surface parameter u at the minimum point* (*u*, *v*) = (0, 0) *is determined by using the* Eqs (2.28) *and* (2.29),

$$\begin{array}{c} \omega_{uu}\left(0,0\right)={\left(m+\varsigma \right)}^{2}(\frac{m-1}{m})(P_{20}-P_{10}+P_{00})=P_{00}^{\left(2,0\right)}. \end{array}$$ (2.34)

*The value of* ***ω***_*uv*_ (0, 0), *the second order partial derivatives of SKBS with respect to the surface parameters* (*u and v*) *at the minimum point* (*u*, *v*) = (0, 0) *is obtained by using the* Eqs (2.30) *and* (2.31),

$$\begin{array}{c} \omega_{uv}\left(0,0\right)=(m+\varsigma )(n+\varsigma )(P_{11}-P_{10}-P_{01}+P_{00})=P_{00}^{\left(1,1\right)}. \end{array}$$ (2.35)

*The value of* ***ω***_*vv*_(0, 0), *the second-order-partial derivative of SKBS with respect to the surface parameter v at the minimum point* (*u*, *v*) = (0, 0) *is determined by using the* Eqs (2.32) *and* (2.33),

$$\begin{array}{c} \omega_{vv}\left(0,0\right)={\left(n+\varsigma \right)}^{2}(\frac{n-1}{n})(P_{02}-2P_{01}+P_{00})=P_{00}^{\left(0,2\right)}. \end{array}$$ (2.36)

The shifted-knots Bézier surface in Minkowski space-$E_{1}^{3}$, represented by $\omega \left(u,v\right)=\sum_{k=0}^{m}\sum_{l=0}^{n}S_{♭,\varsigma }^{k,m}(u)S_{♭,\varsigma }^{l,n}(v)P_{kl}$ using the control-points ${\left\{P_{kl}\right\}}_{k,l=0}^{m,n}$ defined by the Minkowski-inner product, is referred to as the non-degenerate SKBS in Minkowski space-$E_{1}^{3}$. If the normal of the surface has a Minkowski inner product of $\xi_{L}(N,N)=1$, it is known as a timelike SKBS, and if $\xi_{L}(N,N)=-1$, it is referred to as a spacelike SKBS.

## 3 Geometric characteristics of shifted-knots Bézier Surfaces

The following section presents an in-depth analysis of the fundamental coefficients, Gauss-curvature, and mean-curvature for timelike and spacelike shifted-knots Bézier surface in the Minkowski space-$E_{1}^{3}$. The geometric quantities found are used to derive the matrix-form of the shape operator of timelike and spacelike surfaces. The analysis concludes with the presentation of specific, quantitative examples in the next section to demonstrate the effectiveness and applicability of the method. It will provide a clear view of how the method is used in practice and what can be achieved by applying the method.

**Theorem 3.1**. *The first fundamental coefficients E*, *F and G of the timelike or spacelike shifted-knots Bézier surface in the Minkowski space*-$E_{1}^{3}$ *are*

$$\begin{array}{c} E=\xi_{L}(\sum_{k,l=0}^{m-1,n}S_{♭,\varsigma }^{k,m-1}(u)S_{♭,\varsigma }^{l,n}(v)P_{kl}^{\left(1,0\right)},\sum_{k,l=0}^{m-1,n}S_{♭,\varsigma }^{k,m-1}(u)S_{♭,\varsigma }^{l,n}(v)P_{kl}^{\left(1,0\right)}), \end{array}$$ (3.1)

$$\begin{array}{c} F=\xi_{L}(\sum_{k,l=0}^{m-1,n}S_{♭,\varsigma }^{k,m-1}(u)S_{♭,\varsigma }^{l,n}(v)P_{kl}^{\left(1,0\right)},\sum_{k,l=0}^{m,n-1}S_{♭,\varsigma }^{k,m}(u)S_{♭,\varsigma }^{l,n-1}(v)P_{kl}^{\left(0,1\right)}), \end{array}$$ (3.2)

$$\begin{array}{c} G=\xi_{L}(\sum_{k,l=0}^{m,n-1}S_{♭,\varsigma }^{k,m-1}(u)S_{♭,\varsigma }^{l,n-1}(v)P_{kl}^{\left(0,1\right)},\sum_{k,l=0}^{m-1,n}S_{♭,\varsigma }^{k,m}(u)S_{♭,\varsigma }^{l,n-1}(v)P_{kl}^{\left(0,1\right)}). \end{array}$$ (3.3)

*Proof*. The first fundamental coefficient *E* (Eq (2.14)) of SKBS is computed by utilizing the first-order partial derivative ***ω***_*u*_(*u*, *v*) of shifted-knots Bézier surface defined in the Eq (2.22), for the Lorentzian-inner product metric defined in Eq (2.10), is

$$\begin{array}{c} \begin{array}{cc} E & =\xi_{L}\left(\omega_{u}\left(u,v\right),\omega_{u}\left(u,v\right)\right)\\ & ={\left(\sum_{k,l=0}^{m-1,n}S_{♭,\varsigma }^{k,m-1}\left(u\right)S_{♭,\varsigma }^{l,n}\left(v\right)x_{kl}^{\left(1,0\right)}\right)}^{2}+{\left(\sum_{k,l=0}^{m-1,n}S_{♭,\varsigma }^{k,m-1}\left(u\right)S_{♭,\varsigma }^{l,n}\left(v\right)y_{kl}^{\left(1,0\right)}\right)}^{2}-\\ & \;{\left(\sum_{k,l=0}^{m-1,n}S_{♭,\varsigma }^{k,m-1}\left(u\right)S_{♭,\varsigma }^{l,n}\left(v\right)z_{kl}^{\left(1,0\right)}\right)}^{2}\\ & =\xi_{L}\left(\sum_{k,l=0}^{m-1,n}S_{♭,\varsigma }^{k,m-1}\left(u\right)S_{♭,\varsigma }^{l,n}\left(v\right)S_{kl}^{\left(1,0\right)},\sum_{k,l=0}^{m-1,n}S_{♭,\varsigma }^{k,m-1}\left(u\right)S_{♭,\varsigma }^{l,n}\left(v\right)S_{kl}^{\left(1,0\right)}\right). \end{array} \end{array}$$ (3.4)

In a similar manner, the remaining coefficients *F* and *G* of shifted-knots Bézier surface in Minkowski space-$E_{1}^{3}$ are obtained by utilizing the appropriate partial derivatives of SKBS and the defined Lorentzian-inner product metric. These calculations are useful for the subsequent computation of the shape operator of the SKBS in Minkowski space-$E_{1}^{3}$.

**Corollary 3.1.1**. *At the minimum point* (*u*, *v*) = (0, 0), *the coefficients* *E*, *F and G* *of the first-fundamental form of the timelike or spacelike SKBS in the Minkowski space*-$E_{1}^{3}$ *are obtained from the* Eqs (3.1)–(3.3)

$$\begin{array}{c} E=\xi_{L}(P_{00}^{\left(1,0\right)},P_{00}^{\left(1,0\right)}),\;F=\xi_{L}(P_{00}^{\left(1,0\right)},P_{00}^{\left(0,1\right)}),\;G=\xi_{L}(P_{00}^{\left(0,1\right)},P_{00}^{\left(0,1\right)}). \end{array}$$ (3.5)

**Theorem 3.2**. *The Lorentzian-Minkowski metric* *ds*^2^ = *Edu*^2^ + 2*Fdudv* + *dv*^2^ *of the timelike or spacelike SKBS in Minkowski space*-$E_{1}^{3}$ *is given by*

$$\begin{array}{c} \begin{array}{cc} ds^{2} & =\xi_{L}(\sum_{ȷ,l=0}^{m-1,n}S_{♭,\varsigma }^{k,m-1}(u)S_{♭,\varsigma }^{l,n}(v)P_{kl}^{\left(1,0\right)},\sum_{k,l=0}^{m-1,n}S_{♭,\varsigma }^{k,m-1}(u)S_{♭,\varsigma }^{l,n}(v)P_{kl}^{\left(1,0\right)})du^{2}\\ & +2\xi_{L}(\sum_{k,l=0}^{m-1,n}S_{♭,\varsigma }^{k,m-1}(u)S_{♭,\varsigma }^{l,n}(v)P_{kl}^{\left(1,0\right)},\sum_{k,l=0}^{m,n-1}S_{♭,\varsigma }^{k,m}(u)S_{♭,\varsigma }^{l,n-1}(v)P_{kl}^{\left(0,1\right)})dudv\\ & +\xi_{L}(\sum_{k,l=0}^{m,n-1}S_{♭,\varsigma }^{k,m-1}(u)S_{♭,\varsigma }^{l,n-1}(v)P_{kl}^{\left(0,1\right)},\sum_{k,l=0}^{m-1,n}S_{♭,\varsigma }^{k,m}(u)S_{♭,\varsigma }^{l,n-1}(v)P_{kl}^{\left(0,1\right)})dv^{2}. \end{array} \end{array}$$ (3.6)

**Corollary 3.2.1**. *In the Minkowski space*-$E_{1}^{3}$, *the Lorentzian-Minkowski metric of the timelike or spacelike shifted-knots Bézier surface at the minimum point* (*u*, *v*) = (0, 0) *can be obtained from the* Eq (3.6) *and it is given by*

$$\begin{array}{c} ds^{2}=\xi_{L}(P_{00}^{\left(1,0\right)},P_{00}^{\left(1,0\right)})du^{2}+2\xi_{L}(P_{00}^{\left(1,0\right)},P_{00}^{\left(0,1\right)})dudv+\xi_{L}(P_{00}^{\left(0,1\right)},P_{00}^{\left(0,1\right)})dv^{2}. \end{array}$$ (3.7)

**Theorem 3.3** *In the Minkowski space, for the non-degenerate SKBS, we can compute the components of the vector* ***δ*** = ***ω***_*u*_ ∧_*L*_ ***ω***_*v*_ *using* Eq (2.11). *The vector* ***δ*** *is in the direction of normal* **N** *to shifted-knots Bézier surface for its non-degenerate (timelike and spacelike) cases. For the sake of convenience, the components of the vector* ***δ*** *are denoted by* (*δ*1, *δ*_2_, *δ*_3_) *and they are given by*,

$$\begin{array}{c} \begin{array}{cc} \delta_{1} & =\sum_{k,l=0}^{m,n-1}S_{♭,\varsigma }^{k,m}(u)S_{♭,\varsigma }^{l,n-1}(v)y_{kl}^{\left(0,1\right)}\;\sum_{k,l=0}^{m-1,n}S_{♭,\varsigma }^{k,m-1}(u)S_{♭,\varsigma }^{l,n}(v)z_{kl}^{\left(1,0\right)}\\ & -\sum_{k,l=0}^{m-1,n}S_{♭,\varsigma }^{k,m-1}(u)S_{♭,\varsigma }^{l,n}(v)y_{kl}^{\left(1,0\right)}\;\sum_{k,l=0}^{m,n-1}S_{♭,\varsigma }^{k,m}(u)S_{♭,\varsigma }^{l,n-1}(v)z_{kl}^{\left(0,1\right)}, \end{array} \end{array}$$ (3.8)

$$\begin{array}{c} \begin{array}{cc} \delta_{2} & =\sum_{k,l=0}^{m-1,n}S_{♭,\varsigma }^{k,m-1}(u)S_{♭,\varsigma }^{l,n}(v)x_{kl}^{\left(1,0\right)}\;\sum_{k,l=0}^{m,n-1}S_{♭,\varsigma }^{k,m}(u)S_{♭,\varsigma }^{l,n-1}(v)z_{kl}^{\left(0,1\right)}\\ & -\sum_{k,l=0}^{m,n-1}S_{♭,\varsigma }^{k,m}(u)S_{♭,\varsigma }^{l,n-1}(v)x_{kl}^{\left(0,1\right)}\;\sum_{k,l=0}^{m-1,n}S_{♭,\varsigma }^{k,m-1}(u)S_{♭,\varsigma }^{l,n}(v)z_{kl}^{\left(1,0\right)}, \end{array} \end{array}$$ (3.9)

$$\begin{array}{c} \begin{array}{cc} \delta_{3} & =\sum_{k,l=0}^{m-1,n}S_{♭,\varsigma }^{k,m-1}(u)S_{♭,\varsigma }^{l,n}(v)x_{kl}^{\left(1,0\right)}\;\sum_{k,l=0}^{m,n-1}S_{♭,\varsigma }^{k,m}(u)S_{♭,\varsigma }^{l,n-1}(v)y_{kl}^{\left(0,1\right)}\\ & -\sum_{k,l=0}^{m,n-1}S_{♭,\varsigma }^{k,m}(u)S_{♭,\varsigma }^{l,n-1}(v)x_{kl}^{\left(0,1\right)}\;\sum_{k,l=0}^{m-1,n}S_{♭,\varsigma }^{k,m-1}(u)S_{♭,\varsigma }^{l,n}(v)y_{kl}^{\left(1,0\right)}. \end{array} \end{array}$$ (3.10)

**Corollary 3.3.1**. *The components* *δ*_1_, *δ*_2_ *and* *δ*_3_ *of the vector* ***δ*** *as given in* Eqs (3.8)–(3.10) *can be computed at the point* (*u*, *v*) = (0, 0) *for the timelike and spacelike SKBS in Minkowski space*-$E_{1}^{3}$ *and they can be written in the form*,

$$\begin{array}{c} \begin{array}{cc} & \delta_{1}=(y_{00}^{\left(0,1\right)}z_{00}^{\left(1,0\right)}-y_{00}^{\left(1,0\right)}z_{00}^{\left(0,1\right)}),\\ & \delta_{2}=(x_{00}^{\left(1,0\right)}z_{00}^{\left(0,1\right)}-x_{00}^{\left(0,1\right)}z_{00}^{\left(1,0\right)}),\\ & \delta_{3}=(x_{00}^{\left(1,0\right)}y_{00}^{\left(0,1\right)}-x_{00}^{\left(0,1\right)}y_{00}^{\left(1,0\right)}). \end{array} \end{array}$$ (3.11)

**Theorem 3.4**. *The normal vector-field* **N** *on the timelike or spacelike shifted-knots Bézier surface in Minkowski space*-$E_{1}^{3}$ *is defined as follows*,

$$\begin{array}{c} N=\frac{\delta }{\sqrt{-\epsilon \;\xi_{L}\left(\delta ,\delta \right)}}, \end{array}$$ (3.12)

*where ϵ* = −1 *and* *ξ*_*L*_(***δ***, ***δ***) = +1 *for the timelike SKBS, and ϵ* = +1 *and* *ξ*_*L*_(***δ***, ***δ***) = −1 *for the spacelike surface*.

*Proof*. The normal vector **N** of the timelike or spacelike SKBS in Minkowski space-$E_{1}^{3}$ is calculated by utilizing the Lorentzian-cross product (given in the Eq (2.11)), and the Eqs (2.22) and (2.24), and it is given by

$$\begin{array}{c} N=\frac{\omega_{u}\wedge_{L}\omega_{v}}{\left\|\omega_{u}\wedge_{L}\omega_{v}\right\|}=\frac{\sum_{k=0}^{m-1}\sum_{l=0}^{n}S_{♭,\varsigma }^{k,m-1}(u)S_{♭,\varsigma }^{l,n}(v)P_{kl}^{\left(1,0\right)}\wedge_{L}\;\sum_{k=0}^{m}\sum_{l=0}^{n-1}S_{♭,\varsigma }^{k,m}(u)S_{♭,\varsigma }^{l,n-1}(v)P_{kl}^{\left(0,1\right)}}{\left\|\sum_{k=0}^{m-1}\sum_{l=0}^{n}S_{♭,\varsigma }^{k,m-1}(u)S_{♭,\varsigma }^{l,n}(v)P_{kl}^{\left(1,0\right)}\wedge_{L}\;\sum_{k=0}^{m}\sum_{l=0}^{n-1}S_{♭,\varsigma }^{k,m}(u)S_{♭,\varsigma }^{l,n-1}(v)P_{kl}^{\left(0,1\right)}\right\|}. \end{array}$$ (3.13)

SKBS can be represented as either a timelike-surface or a spacelike-surface. The value of *ξ*_*L*_(**N**, **N**) = *ϵ* is used to determine the surface type, where if SKBS is a timelike-surface then *ξ*_*L*_(**N**, **N**) = *ϵ* = +1 and if it is a spacelike-surface, then *ξ*_*L*_(**N**, **N**) = *ϵ* = −1. The norm of ***ω***_*u*_∧_*L*_***ω***_*v*_ in terms of *ϵ* is expressed as $\|\omega_{u}\wedge_{L}\omega \|={\left(|EG-F^{2}|\right)}^{\frac{1}{2}}={\left(-\epsilon (EG-F^{2})\right)}^{\frac{1}{2}}={\left(-\epsilon \;\text{det}(\eta )\right)}^{\frac{1}{2}}$, while taking the norm of ‖***ω***_*u*_∧_*L*_***ω***_*v*_‖ in the Eq (3.13). We can compute now the surface-normal by virtue of the equations, (2.10), (3.8)–(3.10) and the Eq (3.13), as follows

$$\begin{array}{c} N=\frac{\left(\delta_{1},\delta_{2},\delta_{3}\right)}{{\left(|\delta_{1}^{2}+\delta_{2}^{2}-\delta_{3}^{2}|\right)}^{\frac{1}{2}}}=\frac{\left(\delta_{1},\delta_{2},\delta_{3}\right)}{{\left(-\epsilon \left(\delta_{1}^{2}+\delta_{2}^{2}-\delta_{3}^{2}\right)\right)}^{\frac{1}{2}}}=\frac{\delta }{{\left(-\epsilon \;\xi_{L}\left(\delta ,\delta \right)\right)}^{\frac{1}{2}}}. \end{array}$$ (3.14)

**Corollary 3.4.1**. *The normal-vector* **N** *on shifted-knots Bézier surface which can be either timelike or spacelike in the Minkowski space*-$E_{1}^{3}$ *at the min point* (*u*, *v*) = (0, 0) *is calculated using the Lorentzian cross product and other relevant equations, and takes the following form as described in* Eq (3.13),

$$\begin{array}{c} N\left(0,0\right)=\frac{P_{00}^{\left(1,0\right)}\wedge_{L}\;P_{00}^{\left(0,1\right)}}{\left\|P_{00}^{\left(1,0\right)}\wedge_{L}\;P_{00}^{\left(0,1\right)}\right\|}. \end{array}$$ (3.15)

**Theorem 3.5**. *The determinant of the first fundamental-form of the timelike or spacelike SKBS in the Minkowski space*-$E_{1}^{3}$ *can be written in the following form*,

$$\begin{array}{c} \text{det}(\eta )=-\xi_{L}(\delta ,\delta ). \end{array}$$ (3.16)

*Proof*. The determinant of the first-fundamental form of the timelike or spacelike shifted-knots Bézier surface in the Minkowski space-$E_{1}^{3}$ is det(*η*) = *EG* − *F*^2^ and the rearrangement of the terms in it, by utilizing equations (Eqs (3.1)–(3.3)) the following result is obtained,

$$\begin{array}{c} \begin{array}{cc} \text{det}\left(\eta \right) & ={\left(\begin{array}{cc} & \sum_{k,l=0}^{m-1,n}S_{♭,\varsigma }^{k,m-1}\left(u\right)S_{♭,\varsigma }^{l,n}\left(v\right)x_{kl}^{\left(1,0\right)}\;\sum_{k,l=0}^{m,n-1}S_{♭,\varsigma }^{k,m}\left(u\right)S_{♭,\varsigma }^{l,n-1}\left(v\right)y_{kl}^{\left(0,1\right)}-\\ & \sum_{k,l=0}^{m,n-1}S_{♭,\varsigma }^{k,m}\left(u\right)S_{♭,\varsigma }^{l,n-1}\left(v\right)x_{kl}^{\left(0,1\right)}\;\sum_{k,l=0}^{m-1,n}S_{♭,\varsigma }^{k,m-1}\left(u\right)S_{♭,\varsigma }^{l,n}\left(v\right)y_{kl}^{\left(1,0\right)} \end{array}\right)}^{2}\\ & -{\left(\begin{array}{cc} & \sum_{k,l=0}^{m-1,n}S_{♭,\varsigma }^{k,m-1}\left(u\right)S_{♭,\varsigma }^{l,n}\left(v\right)x_{kl}^{\left(1,0\right)}\;\sum_{k,l=0}^{m,n-1}S_{♭,\varsigma }^{k,m}\left(u\right)S_{♭,\varsigma }^{l,n-1}\left(v\right)z_{kl}^{\left(0,1\right)}-\\ & \sum_{k,l=0}^{m,n-1}S_{♭,\varsigma }^{k,m}\left(u\right)S_{♭,\varsigma }^{l,n-1}\left(v\right)x_{kl}^{\left(0,1\right)}\;\sum_{k,l=0}^{m-1,n}S_{♭,\varsigma }^{k,m-1}\left(u\right)S_{♭,\varsigma }^{l,n}\left(v\right)z_{kl}^{\left(1,0\right)} \end{array}\right)}^{2}\\ & -{\left(\begin{array}{cc} & \sum_{k,l=0}^{m-1,n}S_{♭,\varsigma }^{k,m-1}\left(u\right)S_{♭,\varsigma }^{l,n}\left(v\right)y_{kl}^{\left(1,0\right)}\;\sum_{k,l=0}^{m,n-1}S_{♭,\varsigma }^{k,m}\left(u\right)S_{♭,\varsigma }^{l,n-1}\left(v\right)z_{kl}^{\left(0,1\right)}-\\ & \sum_{k,l=0}^{m,n-1}S_{♭,\varsigma }^{k,m}\left(u\right)S_{♭,\varsigma }^{l,n-1}\left(v\right)y_{kl}^{\left(0,1\right)}\;\sum_{k,l=0}^{m-1,n}S_{♭,\varsigma }^{k,m-1}\left(u\right)S_{♭,\varsigma }^{l,n}\left(v\right)z_{kl}^{\left(1,0\right)} \end{array}\right)}^{2}. \end{array} \end{array}$$ (3.17)

and as a consequence we obtain the resulting Eq (3.16).

**Corollary 3.5.1**. *Using the* Eqs (3.5) *and* (3.16), det(*η*), *the determinant of the first-fundamental form is as follows*,

$$\begin{array}{c} \text{det}(\eta )=EG-F^{2}=\xi_{L}(P_{00}^{\left(1,0\right)},P_{00}^{\left(1,0\right)})\xi_{L}(P_{00}^{\left(0,1\right)},P_{00}^{\left(0,1\right)})-\xi_{L}^{2}(P_{00}^{\left(1,0\right)},P_{00}^{\left(0,1\right)}), \end{array}$$ (3.18)

*and after a little simplification, the* Eq (3.18) *can be written as*,

$$\begin{array}{c} \text{det}(\eta )=-(\delta_{1}^{2}+\delta_{2}^{2}-\delta_{3}^{2})=-\xi_{L}(\delta ,\delta ). \end{array}$$ (3.19)

**Theorem 3.6**. *The coefficients of the second fundamental form of the timelike or spacelike shifted-knots Bézier surface are*

$$\begin{array}{c} e={\left(-\epsilon \;\xi_{L}\left(\delta ,\delta \right)\right)}^{-\frac{1}{2}}\;\xi_{L}\left(\sum_{k=0}^{m-2}\sum_{l=0}^{n}S_{♭,\varsigma }^{k,m-2}\left(u\right)S_{♭,\varsigma }^{l,n}\left(v\right)P_{kl}^{\left(2,0\right)},\delta \right), \end{array}$$ (3.20)

$$\begin{array}{c} f={\left(-\epsilon \;\xi_{L}\left(\delta ,\delta \right)\right)}^{-\frac{1}{2}}\;\xi_{L}\left(\sum_{k=0}^{m-1}\sum_{l=0}^{n-1}S_{♭,\varsigma }^{k,m-1}\left(u\right)S_{♭,\varsigma }^{l,n-1}\left(v\right)P_{kl}^{\left(1,1\right)},\delta \right), \end{array}$$ (3.21)

$$\begin{array}{c} g={\left(-\epsilon \;\xi_{L}\left(\delta ,\delta \right)\right)}^{-\frac{1}{2}}\;\xi_{L}\left(\sum_{k=0}^{m}\sum_{l=0}^{n-2}S_{♭,\varsigma }^{k,m}\left(u\right)S_{♭,\varsigma }^{l,n-2}\left(v\right)P_{kl}^{\left(0,2\right)},\delta \right). \end{array}$$ (3.22)

*Proof*. The second fundamental coefficients of the timelike or spacelike shifted-knots Bézier surface can be calculated using ***ω***(*u*, *v*), $e=\xi_{L}\left(\omega_{uu},N\right)$, $f=\xi_{L}\left(\omega_{uv},N\right)$ and $g=\xi_{L}\left(\omega_{vv},N\right)$. In particular, the coefficient *e* of the second fundamental-form, for the second-order partial derivative given by the Eq (2.28) and the normal-vector field **N** given by the Eq (3.13)) is as follows,

$$\begin{array}{c} \begin{array}{cc} e= & {-(\|\sum_{k=0}^{m-1}\sum_{l=0}^{n}S_{♭,\varsigma }^{k,m-1}(u)S_{♭,\varsigma }^{l,n}(v)P_{kl}^{\left(1,0\right)}\wedge_{L}\;\sum_{k=0}^{m}\sum_{l=0}^{n-1}S_{♭,\varsigma }^{k,m}(u)S_{♭,\varsigma }^{l,n-1}(v)P_{kl}^{\left(0,1\right)}\|)}^{-1}\\ & \text{det}(\sum_{k=0}^{m-2}\sum_{l=0}^{n}S_{♭,\varsigma }^{k,m-2}(u)S_{♭,\varsigma }^{l,n}(v)P_{kl}^{\left(2,0\right)},\sum_{k=0}^{m-1}\sum_{l=0}^{n}S_{♭,\varsigma }^{k,m-1}(u)S_{♭,\varsigma }^{l,n}(v)P_{kl}^{\left(1,0\right)},\\ & \sum_{k=0}^{m}\sum_{l=0}^{n-1}S_{♭,\varsigma }^{k,m}(u)S_{♭,\varsigma }^{l,n-1}(v)P_{kl}^{\left(0,1\right)}), \end{array} \end{array}$$ (3.23)

and for the Lorentzian cross product of vectors given by the Eq (2.11), it follows that

$$\begin{array}{c} \begin{array}{cc} e= & \frac{1}{\sqrt{\left|\delta_{1}^{2}+\delta_{2}^{2}-\delta_{3}^{2}\right|}}(-\sum_{k=0}^{m-2}\sum_{l=0}^{n}S_{♭,\varsigma }^{k,m-2}(u)S_{♭,\varsigma }^{l,n}(v)x_{kl}^{\left(2,0\right)}\left(-\delta_{1}\right)-\\ & \sum_{k=0}^{m-2}\sum_{l=0}^{n}S_{♭,\varsigma }^{k,m-2}(u)S_{♭,\varsigma }^{l,n}(v)y_{kl}^{\left(2,0\right)}\left(-\delta_{2}\right)-\sum_{k=0}^{m-2}\sum_{l=0}^{n}S_{♭,\varsigma }^{k,m-2}(u)S_{♭,\varsigma }^{l,n}(v)x_{kl}^{\left(2,0\right)}\delta_{3}), \end{array} \end{array}$$ (3.24)

which can be simplified as follows,

$$\begin{array}{c} \begin{array}{cc} e= & \frac{1}{\sqrt{-\epsilon (\delta_{1}^{2}+\delta_{2}^{2}-\delta_{3}^{2})}}(\sum_{k=0}^{m-2}\sum_{l=0}^{n}S_{♭,\varsigma }^{k,m-2}(u)S_{♭,\varsigma }^{l,n}(v)x_{kl}^{\left(2,0\right)}\delta_{1}+\\ & \sum_{k=0}^{m-2}\sum_{l=0}^{n}S_{♭,\varsigma }^{k,m-2}(u)S_{♭,\varsigma }^{l,n}(v)y_{kl}^{\left(2,0\right)}\delta_{2}-\sum_{k=0}^{m-2}\sum_{l=0}^{n}S_{♭,\varsigma }^{k,m-2}(u)S_{♭,\varsigma }^{l,n}(v)x_{kl}^{\left(2,0\right)}\delta_{3}). \end{array} \end{array}$$ (3.25)

Similarly, the coefficient *f* and *g* of the second-fundamental form can be obtained by using the Theorem (2.3).

**Corollary 3.6.1**. *The second-fundamental coefficients e*, *f*, *g* *of the timelike or spacelike shifted-knots Bézier surface in the Minkowski space*-$E_{1}^{3}$, *at the min point* (*u*, *v*) = (0, 0) *can be obtained from the* Eqs (3.20)–(3.22), *where* ***δ*** = (*δ*_1_, *δ*_2_, *δ*_3_)

$$\begin{array}{c} e=\frac{\xi_{L}(P_{kl}^{\left(2,0\right)},\delta )}{\sqrt{-\epsilon \;\xi_{L}(\delta ,\delta )}},\;f=\frac{\xi_{L}(P_{kl}^{\left(1,1\right)},\;\delta )}{\sqrt{-\epsilon \;\xi_{L}(\delta ,\delta )}},\;g=\frac{\xi_{L}(P_{kl}^{\left(0,2\right)},\;\delta )}{\sqrt{-\epsilon \;\xi_{L}(\delta ,\delta )}}, \end{array}$$ (3.26)

*and* *δ*_1_, *δ*_2_ *and* *δ*_3_ *are given in the* Eq (3.11).

**Theorem 3.7**. *The Gauss-curvature and the mean curvature of the timelike or spacelike SKBS in the Minkowski space*-$E_{1}^{3}$ *are*

$$\begin{array}{c} \begin{array}{cc} K= & \frac{\epsilon }{\xi_{L}^{2}(\delta ,\delta )}(\xi_{L}(\sum_{k=0}^{m-2}\sum_{l=0}^{n}S_{♭,\varsigma }^{k,m-2}(u)S_{♭,\varsigma }^{l,n}(v)P_{kl}^{\left(2,0\right)},\delta )\xi_{L}(\sum_{k=0}^{m}\sum_{l=0}^{n-2}S_{♭,\varsigma }^{k,m}(u)S_{♭,\varsigma }^{l,n-2}(v)P_{kl}^{\left(0,2\right)},\delta )-\\ & \xi_{L}^{2}(\sum_{k=0}^{m-1}\sum_{l=0}^{n-1}S_{♭,\varsigma }^{k,m-1}(u)S_{♭,\varsigma }^{l,n-1}(v)P_{kl}^{\left(1,1\right)},\delta )), \end{array} \end{array}$$ (3.27)

*and*

$$\begin{array}{c} \begin{array}{cc} H= & -\frac{\epsilon }{2\sqrt{\epsilon \xi_{L}^{3}{\left(\delta ,\delta \right)}_{L}}}(\xi_{L}(\sum_{k,l=0}^{m-2,n}S_{♭,\varsigma }^{k,m-2}(u)S_{♭,\varsigma }^{l,n}(v)P_{kl}^{\left(2,0\right)},\delta )\times \\ & \xi_{L}(\sum_{k,l=0}^{m,n-1}S_{♭,\varsigma }^{k,m}(u)S_{♭,\varsigma }^{l,n-1}(v)P_{kl}^{\left(0,1\right)},\sum_{k,l=0}^{m,n-1}S_{♭,\varsigma }^{k,m}(u)S_{♭,\varsigma }^{l,n-1}(v)P_{kl}^{\left(0,1\right)})-\\ & 2\xi_{L}(\sum_{k,l=0}^{m-1,n-1}S_{♭,\varsigma }^{k,m-1}(u)S_{♭,\varsigma }^{l,n-1}(v)P_{kl}^{\left(1,1\right)},\delta )\times \\ & \xi_{L}(\sum_{k,l=0}^{m-1,n}S_{♭,\varsigma }^{k,m-1}(u)S_{♭,\varsigma }^{l,n}(v)P_{kl}^{\left(1,0\right)},\sum_{k,l=0}^{m,n-1}S_{♭,\varsigma }^{k,m}(u)S_{♭,\varsigma }^{l,n-1}(v)P_{kl}^{\left(0,1\right)})+\\ & \xi_{L}(\sum_{k,l=0}^{m,n-2}S_{♭,\varsigma }^{k,m}(u)S_{♭,\varsigma }^{n-2}(v)P_{kl}^{\left(0,2\right)},\delta )\times \\ & \xi_{L}(\sum_{k,l=0}^{m-1,n}S_{♭,\varsigma }^{k,m-1}(u)S_{♭,\varsigma }^{l,n}(v)P_{kl}^{\left(1,0\right)},\sum_{k,l=0}^{m-1,n}S_{♭,\varsigma }^{k,m-1}(u)S_{♭,\varsigma }^{l,n}(v)P_{kl}^{\left(1,0\right)})). \end{array} \end{array}$$ (3.28)

*Proof*. The Gauss-curvature and the mean-curvature of the timelike or spacelike SKBS in the Minkowski space-$E_{1}^{3}$ can be obtained by utilizing the Theorem (3.1) and the Theorem (3.6). The Gauss-curvature $K=\epsilon (\frac{eg-f^{2}}{EG-F^{2}})$ of SKBS in the Minkowski space-$E_{1}^{3}$ is given by

$$\begin{array}{c} \begin{array}{cc} K= & \frac{\epsilon }{-\xi_{L}\left(\delta ,\delta \right)}(\frac{\xi_{L}(\sum_{k,l=0}^{m-2,n}S_{♭,\varsigma }^{k,m-2}(u)S_{♭,\varsigma }^{l,n}(v)P_{kl}^{\left(2,0\right)},\delta )}{\sqrt{-\epsilon \;\xi_{L}\left(\delta ,\delta \right)}}\times \\ & \frac{\xi_{L}(\sum_{k,l=0}^{m,n-2}S_{♭,\varsigma }^{k,m-2}(u)S_{♭,\varsigma }^{l,n-2}(v)P_{kl}^{\left(0,2\right)},\delta )}{\sqrt{-\epsilon \;\xi_{L}\left(\delta ,\delta \right)}}-\frac{\xi_{L}^{2}(\sum_{k,l=0}^{m-1,n-1}S_{♭,\varsigma }^{k,m-1}(u)S_{♭,\varsigma }^{l,n-1}(v)P_{kl}^{\left(1,1\right)},\delta )}{{\left(\sqrt{-\epsilon \;\xi_{L}\left(\delta ,\delta \right)}\right)}^{2}}). \end{array} \end{array}$$ (3.29)

Similarly, we can find the desired expression for mean-curvature *H* of shifted-knots Bézier surface as stated in the Eq (3.28) in the Minkowski space-$E_{1}^{3}$.

**Corollary 3.7.1**. *The Gauss-curvature of the timelike or spacelike SKBS at the minimum point* (*u*, *v*) = (0, 0) *in Minkowski space*-$E_{1}^{3}$ *is determined by utilizing the* Eq (3.27)

$$\begin{array}{c} K=\frac{-\epsilon }{\xi_{L}^{2}\left(\delta ,\delta \right)}(\xi_{L}(P_{00}^{\left(2,0\right)},\delta )\xi_{L}(P_{00}^{\left(0,2\right)},\delta )-\xi_{L}^{2}(P_{00}^{\left(1,1\right)},\delta )). \end{array}$$ (3.30)

*The mean-curvature of the timelike or spacelike SKBS at the point* (*u*, *v*) = (0, 0) *is obtained by the* Eq (3.28) *in Minkowski space*-$E_{1}^{3}$

$$\begin{array}{c} \begin{array}{cc} H= & \frac{-\epsilon }{2\sqrt{-\epsilon \;\xi_{L}^{3}\left(\delta ,\delta \right)}}(\xi_{L}(P_{00}^{\left(2,0\right)},\delta )\xi_{L}(P_{00}^{\left(0,1\right)},P_{00}^{\left(0,1\right)})-2\xi_{L}(P_{00}^{\left(1,1\right)},\delta )\xi_{L}(P_{00}^{\left(1,0\right)},P_{00}^{\left(0,1\right)})+\\ & \xi_{L}(P_{00}^{\left(0,2\right)},\delta )\xi_{L}(P_{00}^{\left(1,0\right)},P_{00}^{\left(1,0\right)})). \end{array} \end{array}$$ (3.31)

**Theorem 3.8**. *The coefficients of the matrix* $\lambda =[\begin{array}{cc} \lambda_{11} & \lambda_{12}\\ \lambda_{21} & \lambda_{22} \end{array}]$ *corresponding to the shape-operator of the timelike or spacelike SKBS in the Minkowski space*-$E_{1}^{3}$ *are*

$$\begin{array}{c} \begin{array}{cc} \lambda_{11}= & \frac{-1}{\sqrt{-\epsilon \;\xi_{L}^{3}\left(\delta ,\delta \right)}}(\xi_{L}(\sum_{k,l=0}^{m-2,n}S_{♭,\varsigma }^{k,m-2}(u)S_{♭,\varsigma }^{l,n}(v)P_{kl}^{\left(2,0\right)},\delta )\times \\ & \xi_{L}(\sum_{k,l=0}^{m,n-1}S_{♭,\varsigma }^{k,m}(u)S_{♭,\varsigma }^{l,n-1}(v)P_{kl}^{\left(0,1\right)},\sum_{k,l=0}^{m,n-1}S_{♭,\varsigma }^{k,m}(u)S_{♭,\varsigma }^{l,n-1}(v)P_{kl}^{\left(0,1\right)}))+\\ & \frac{1}{\sqrt{-\epsilon \;\xi_{L}^{3}\left(\delta ,\delta \right)}}(\xi_{L}(\sum_{k,l=0}^{m-1,n-1}S_{♭,\varsigma }^{k,m-1}(u)S_{♭,\varsigma }^{l,n-1}(v)P_{kl}^{\left(1,1\right)},\delta )\times \\ & \xi_{L}(\sum_{k,l=0}^{m-1,n}S_{♭,\varsigma }^{k,m-1}(u)S_{♭,\varsigma }^{n}(v)P_{kl}^{\left(1,0\right)},\sum_{k,l=0}^{m,n-1}S_{♭,\varsigma }^{k,m}(u)S_{♭,\varsigma }^{l,n-1}(v)P_{kl}^{\left(0,1\right)})), \end{array} \end{array}$$ (3.32)

$$\begin{array}{c} \begin{array}{cc} \lambda_{12}= & \frac{-1}{\sqrt{-\epsilon \;\xi_{L}^{3}\left(\delta ,\delta \right)}}(\xi_{L}(\sum_{k,l=0}^{m-1,n-1}S_{♭,\varsigma }^{k,m-1}(u)S_{♭,\varsigma }^{l,n-1}(v)P_{kl}^{\left(1,1\right)},\delta )\times \\ & \xi_{L}(\sum_{k,l=0}^{m,n-1}S_{♭,\varsigma }^{k,m}(u)S_{♭,\varsigma }^{l,n-1}(v)P_{kl}^{\left(0,1\right)},\sum_{k,l=0}^{m,n-1}S_{♭,\varsigma }^{k,m}(u)S_{♭,\varsigma }^{l,n-1}(v)P_{kl}^{\left(0,1\right)}))+\\ & \frac{1}{\sqrt{-\epsilon \;\xi_{L}^{3}\left(\delta ,\delta \right)}}(\xi_{L}(\sum_{k,l=0}^{m,n-2}S_{♭,\varsigma }^{k,m}(u)S_{♭,\varsigma }^{l,n-2}(v)P_{kl}^{\left(0,2\right)},\delta )\times \\ & \xi_{L}(\sum_{k,l=0}^{m-1,n}S_{♭,\varsigma }^{k,m}(u)S_{♭,\varsigma }^{l,n}(v)P_{kl}^{\left(1,0\right)},\sum_{k,l=0}^{m,n-1}S_{♭,\varsigma }^{k,m}(u)S_{♭,\varsigma }^{l,n-1}(v)P_{kl}^{\left(0,1\right)})), \end{array} \end{array}$$ (3.33)

$$\begin{array}{c} \begin{array}{cc} \lambda_{21}= & \frac{1}{\sqrt{-\epsilon \;\xi_{L}^{3}\left(\delta ,\delta \right)}}(\xi_{L}(\sum_{k,l=0}^{m-2,n}S_{♭,\varsigma }^{k,m-2}(u)S_{♭,\varsigma }^{l,n}(v)P_{kl}^{\left(2,0\right)},\delta )\times \\ & \xi_{L}(\sum_{k,l=0}^{m-1,n}S_{♭,\varsigma }^{k,m-1}(u)S_{♭,\varsigma }^{l,n}(v)P_{kl}^{\left(1,0\right)},\sum_{k,l=0}^{m,n-1}S_{♭,\varsigma }^{k,m}(u)S_{♭,\varsigma }^{l,n-1}(v)P_{kl}^{\left(0,1\right)}))-\\ & \frac{1}{\sqrt{-\epsilon \;\xi_{L}^{3}\left(\delta ,\delta \right)}}(\xi_{L}(\sum_{k,l=0}^{m-1,n-1}S_{♭,\varsigma }^{k,m-1}(u)S_{♭,\varsigma }^{l,n-1}(v)P_{kl}^{\left(1,1\right)},\delta )\times \\ & \xi_{L}(\sum_{k,l=0}^{m-1,n}S_{♭,\varsigma }^{k,m-1}(u)S_{♭,\varsigma }^{l,n}(v)P_{kl}^{\left(1,0\right)},\sum_{k,l=0}^{m-1,n}S_{♭,\varsigma }^{k,m-1}(u)S_{♭,\varsigma }^{l,n}(v)P_{kl}^{\left(1,0\right)})), \end{array} \end{array}$$ (3.34)

$$\begin{array}{c} \begin{array}{cc} \lambda_{22}= & \frac{1}{\sqrt{-\epsilon \;\xi_{L}^{3}\left(\delta ,\delta \right)}}(\xi_{L}(\sum_{k,l=0}^{m-1,n-1}S_{♭,\varsigma }^{k,m-1}(u)S_{♭,\varsigma }^{l,n-1}(v)P_{kl}^{\left(1,1\right)},\delta )\times \\ & \xi_{L}(\sum_{k,l=0}^{m-1,n}S_{♭,\varsigma }^{k,m-1}(u)S_{♭,\varsigma }^{l,n}(v)P_{kl}^{\left(1,0\right)},\sum_{k,l=0}^{m,n-1}S_{♭,\varsigma }^{k,m}(u)S_{♭,\varsigma }^{l,n-1}(v)P_{kl}^{\left(0,1\right)}))-\\ & \frac{1}{\sqrt{-\epsilon \;\xi_{L}^{3}\left(\delta ,\delta \right)}}(\xi_{L}(\sum_{k,l=0}^{m,n-2}S_{♭,\varsigma }^{k,m}(u)S_{♭,\varsigma }^{l,n-2}(v)P_{kl}^{\left(0,2\right)},\delta )\times \\ & \xi_{L}(\sum_{k,l=0}^{m-1,n}S_{♭,\varsigma }^{k,m-1}(u)S_{♭,\varsigma }^{l,n}(v)P_{kl}^{\left(1,0\right)},\sum_{k,l=0}^{m-1,n}S_{♭,\varsigma }^{k,m-1}(u)S_{♭,\varsigma }^{l,n}(v)P_{kl}^{\left(1,0\right)})). \end{array} \end{array}$$ (3.35)

*Proof*. The coefficients of the matrix corresponding to the shape-operator of the timelike or spacelike SKBS in the Minkowski space-$E_{1}^{3}$ can be obtained from the Eq (2.18) and one of these coefficients is $\lambda_{11}=\frac{eG-fF}{EG-F^{2}}$. Substituting the Eqs (3.2), (3.3), (3.16), (3.20) and (3.21) for the corresponding fundamental coefficients in this equation we find,

$$\begin{array}{c} \begin{array}{cc} \lambda_{11}= & \frac{1}{\sqrt{-\epsilon \xi_{L}^{3}\left(\delta ,\delta \right)}}(\xi_{L}(\sum_{k,l=0}^{m-2,n}S_{♭,\varsigma }^{k,m-2}(u)S_{♭,\varsigma }^{l,n}(v)P_{kl}^{\left(2,0\right)},\delta )\times \\ & \xi_{L}(\sum_{k,l=0}^{m,n-1}S_{♭,\varsigma }^{k,m}(u)S_{♭,\varsigma }^{l,n-1}(v)P_{kl}^{\left(0,1\right)},\sum_{k,l=0}^{m,n-1}S_{♭,\varsigma }^{k,m}(u)S_{♭,\varsigma }^{l,n-1}(v)P_{kl}^{\left(0,1\right)})+\\ & \xi_{L}(\sum_{k,l=0}^{m-1,n-1}S_{♭,\varsigma }^{k,m-1}(u)S_{♭,\varsigma }^{l,n-1}(v)P_{kl}^{\left(1,1\right)},\delta )\times \\ & \xi_{L}(\sum_{k,l=0}^{m-1,n}S_{♭,\varsigma }^{k,m-1}(u)S_{♭,\varsigma }^{l,n}(v)P_{kl}^{\left(1,0\right)},\sum_{k,l=0}^{m,n-1}S_{♭,\varsigma }^{k,m}(u)S_{♭,\varsigma }^{l,n-1}(v)P_{kl}^{\left(0,1\right)})), \end{array} \end{array}$$ (3.36)

which reduces to the Eq (3.32) after a little simplification. Similarly, other matrix-components λ_12_, λ_21_ and λ_22_ of the timelike or spacelike SKBS in the Minkowski space-$E_{1}^{3}$ can be determined as given in the Eqs (3.33)–(3.35).

**Corollary 3.8.1**. *The coefficients of the matrix* λ, *which corresponds to the shape-operator of the timelike or spacelike SKBS, at the point* (*u*, *v*) = (0, 0) *can be computed using* Eqs (3.32)–(3.35) *of the Theorem (3.8) and they are*

$$\begin{array}{c} \lambda_{11}=\frac{-1}{\sqrt{-\epsilon \;\xi_{L}^{3}(\delta ,\delta )}}(\xi_{L}(P_{00}^{\left(2,0\right)},\delta )\xi_{L}(P_{00}^{\left(0,1\right)},P_{00}^{\left(0,1\right)})-\xi_{L}(P_{00}^{\left(1,1\right)},\delta )\xi_{L}(P_{00}^{\left(1,0\right)},P_{00}^{\left(0,1\right)})), \end{array}$$ (3.37)

$$\begin{array}{c} \lambda_{12}=\frac{-1}{\sqrt{-\epsilon \;\xi_{L}^{3}(\delta ,\delta )}}(\xi_{L}(P_{00}^{\left(1,1\right)},\delta )\xi_{L}(P_{00}^{\left(0,1\right)},P_{00}^{\left(0,1\right)})-\xi_{L}(P_{00}^{\left(0,2\right)},\delta )\xi_{L}(P_{00}^{\left(1,0\right)},P_{00}^{\left(0,1\right)})), \end{array}$$ (3.38)

$$\begin{array}{c} \lambda_{21}=\frac{1}{\sqrt{-\epsilon \;\xi_{L}^{3}(\delta ,\delta )}}(\xi_{L}(P_{00}^{\left(2,0\right)},\delta )\xi_{L}(P_{00}^{\left(1,0\right)},P_{00}^{\left(0,1\right)})-\xi_{L}(P_{00}^{\left(1,1\right)},\delta )\xi_{L}(P_{00}^{\left(1,0\right)},P_{00}^{\left(1,0\right)})), \end{array}$$ (3.39)

$$\begin{array}{c} \lambda_{22}=\frac{1}{\sqrt{-\epsilon \;\xi_{L}^{3}(\delta ,\delta )}}(\xi_{L}(P_{00}^{\left(1,1\right)},\delta )\xi_{L}(P_{00}^{\left(1,0\right)},P_{00}^{\left(0,1\right)})-\xi_{L}(P_{00}^{\left(0,2\right)},\delta )\xi_{L}(P_{00}^{\left(1,0\right)},P_{00}^{\left(0,1\right)})). \end{array}$$ (3.40)

**Theorem 3.9**. *The Gauss and the mean-curvature of the timelike and spacelike SKBS in the Minkowski space*-$E_{1}^{3}$ *can be computed by utilizing shape operator, through the following equations*,

$$\begin{array}{c} \begin{array}{cc} K= & -\epsilon (\xi_{L}(\sum_{k,l=0}^{m-2,n}S_{♭,\varsigma }^{k,m-2}(u)S_{♭,\varsigma }^{l,n}(v)P_{kl}^{\left(2,0\right)},\delta )\xi_{L}(\sum_{k,l=0}^{m,n-2}S_{♭,\varsigma }^{k,m}(u)S_{♭,\varsigma }^{l,n-2}(v)P_{kl}^{\left(0,2\right)},\delta )\\ & -\xi_{L}{\left(\sum_{k,l=0}^{m-1,n-1}S_{♭,\varsigma }^{k,m-1}(u)S_{♭,\varsigma }^{l,n-1}(v)P_{kl}^{\left(1,1\right)},\delta \right)}^{2}){\left(\xi_{L}^{2}\left(\delta ,\delta \right)\right)}^{-1}, \end{array} \end{array}$$ (3.41)

$$\begin{array}{c} \begin{array}{cc} H= & \frac{-\epsilon }{2\sqrt{\epsilon \;\xi_{L}^{3}{\left(\delta ,\delta \right)}_{L}}}(\xi_{L}(\sum_{k,l=0}^{m-2,n}S_{♭,\varsigma }^{k,m-2}(u)S_{♭,\varsigma }^{l,n}(v)P_{kl}^{\left(2,0\right)},\delta )\times \\ & \xi_{L}(\sum_{k,l=0}^{m,n-1}S_{♭,\varsigma }^{k,m}(u)S_{♭,\varsigma }^{l,n-1}(v)P_{kl}^{\left(0,1\right)},\sum_{k,l=0}^{m,n-1}S_{♭,\varsigma }^{k,m}(u)S_{♭,\varsigma }^{l,n-1}(v)P_{kl}^{\left(0,1\right)}))+\\ & \frac{\epsilon }{\sqrt{\epsilon \;\xi_{L}^{3}{\left(\delta ,\delta \right)}_{L}}}(\xi_{L}(\sum_{k,l=0}^{m-1,n-1}S_{♭,\varsigma }^{k,m-1}(u)S_{♭,\varsigma }^{l,n-1}(v)P_{kl}^{\left(1,1\right)},\delta )\times \\ & \xi_{L}(\sum_{k,l=0}^{m-1,n}S_{♭,\varsigma }^{k,m-1}(u)S_{♭,\varsigma }^{l,n}(v)P_{kl}^{\left(1,0\right)},\sum_{k,l=0}^{m,n-1}S_{♭,\varsigma }^{k,m}(u)S_{♭,\varsigma }^{l,n-1}(v)P_{kl}^{\left(0,1\right)}))-\\ & \frac{\epsilon }{2\sqrt{\epsilon \;\xi_{L}^{3}{\left(\delta ,\delta \right)}_{L}}}(\xi_{L}(\sum_{k,l=0}^{m,n-2}S_{♭,\varsigma }^{k,m}(u)S_{♭,\varsigma }^{n-2}(v)P_{kl}^{\left(0,2\right)},\delta )\times \\ & \xi_{L}(\sum_{k,l=0}^{m-1,n}S_{♭,\varsigma }^{k,m-1}(u)S_{♭,\varsigma }^{l,n}(v)P_{kl}^{\left(1,0\right)},\sum_{k,l=0}^{m-1,n}S_{♭,\varsigma }^{k,m-1}(u)S_{♭,\varsigma }^{l,n}(v)P_{kl}^{\left(1,0\right)})). \end{array} \end{array}$$ (3.42)

*Proof*. The Gaussian and mean curvature of the timelike or spacelike SKBS in the Minkowski space-$E_{1}^{3}$ can be determined using the coefficients of the matrix corresponding to shape-operator. The Gaussian curvature (Eq (2.19)) of the timelike and spacelike SKBS is

$$\begin{array}{c} K=\epsilon \;\text{det}\left(\lambda \right)=\epsilon (\lambda_{11}\;\lambda_{22}-\lambda_{12}\;\lambda_{21}), \end{array}$$ (3.43)

plugging the values of the matrix coefficients λ_*jk*_ (*j*, *k* = 1, 2) from the Eqs (3.32)–(3.35) into Eq (3.43), we have

$$\begin{array}{c} \begin{array}{cc} K & =\frac{\epsilon (\delta_{3}^{2}-\delta_{2}^{2}-\delta_{1}^{2})}{\xi_{L}^{3}(\delta ,\delta )}(-\xi_{L}{\left(\sum_{k,l=0}^{m-1,n-1}S_{♭,\varsigma }^{k,m-1}(u)S_{♭,\varsigma }^{l,n-1}(v)P_{kl}^{\left(1,1\right)},\delta \right)}^{2}+\\ & \xi_{L}(\sum_{k,l=0}^{m-2,n}S_{♭,\varsigma }^{k,m-2}(u)S_{♭,\varsigma }^{l,n}(v)P_{kl}^{\left(2,0\right)},\delta )\xi_{L}(\sum_{k,l=0}^{m,n-2}S_{♭,\varsigma }^{k,m}(u)S_{♭,\varsigma }^{l,n-2}(v)P_{kl}^{\left(0,2\right)},\delta )). \end{array} \end{array}$$ (3.44)

The above Eq (3.44) can be cast in the form using Eq (2.10), as follows,

$$\begin{array}{c} \begin{array}{cc} K= & \frac{-\epsilon \;\xi_{L}(\delta ,\delta )}{\xi_{L}^{3}(\delta ,\delta )}(\xi_{L}(\sum_{k,l=0}^{m-2,n}S_{♭,\varsigma }^{k,m-2}(u)S_{♭,\varsigma }^{l,n}(v)P_{kl}^{\left(2,0\right)},\delta )\xi_{L}(\sum_{k,l=0}^{m,n-2}S_{♭,\varsigma }^{k,m}(u)S_{♭,\varsigma }^{l,n-2}(v)P_{kl}^{\left(0,2\right)},\delta )-\\ & \xi_{L}{\left(\sum_{k,l=0}^{m-1,n-1}S_{♭,\varsigma }^{k,m-1}(u)S_{♭,\varsigma }^{l,n-1}(v)P_{kl}^{\left(1,1\right)},\delta \right)}^{2}), \end{array} \end{array}$$ (3.45)

which then easily simplifies to the desired equation for the Gaussian curvature of the surface as given by Eq (3.41). In the similar way, the mean-curvature $H=\frac{\epsilon }{2}trac\left(\lambda \right)=\frac{\epsilon }{2}(\lambda_{11}+\lambda_{22})$ can be computed using the Eqs (3.32) and (3.35).

**Corollary 3.9.1**. *The Gaussian and mean curvatures of the timelike or spacelike SKBS in Minkowski space*-$E_{1}^{3}$ *can be calculated by using the coefficients of the shape-operator at the minimum point* (*u*, *v*) = (0, 0). *This is accomplished by applying the* Eqs (3.41) *and* (3.42) *and they are*

$$\begin{array}{c} K=\frac{-\epsilon (\xi_{L}(P_{00}^{\left(2,0\right)},\delta )\xi_{L}(P_{00}^{\left(0,2\right)},\delta )-\xi_{L}^{2}(P_{00}^{\left(1,1\right)},\delta ))}{\xi_{L}^{2}(\delta ,\delta )}, \end{array}$$ (3.46)

*and*

$$\begin{array}{c} \begin{array}{cc} H= & \frac{-\epsilon }{2\sqrt{-\epsilon \;\xi_{L}^{3}(\delta ,\delta )}}(\xi_{L}(P_{00}^{\left(2,0\right)},\delta )\xi_{L}(P_{00}^{\left(0,1\right)},P_{00}^{\left(0,1\right)})-2\xi_{L}(P_{00}^{\left(1,1\right)},\delta )\xi_{L}(P_{00}^{\left(1,0\right)},P_{00}^{\left(0,1\right)})+\\ & \xi_{L}(P_{00}^{\left(0,2\right)},\delta )\xi_{L}(P_{00}^{\left(1,0\right)},P_{00}^{\left(1,0\right)})). \end{array} \end{array}$$ (3.47)

## 4 Shape operator of shifted-knots Bézier surfaces

In this section, we illustrate the scheme outlined in the previous section (3) for finding the geometric quantities of timelike and spacelike bi-quadratic and bi-cubic shifted-knots Bézier surfaces. This is achieved by presenting detailed discussions of four special cases for *m* = *n* = 2 and *m* = *n* = 3 for biquadratic and bicubic shifted-knots Bézier surfaces, respectively. Numeric examples are provided to show the computation of mean curvature, Gaussian curvature and the shape-operator of the non-degenerate cases of these surfaces for various values of the shape parameters, ♭ and ς. The aim is to demonstrate the applicability of the method outlined in section (3) to finding the shape-operator of timelike and spacelike SKBS (Eq (2.6)) in the Minkowski space-$E_{1}^{3}$.

**Case-1**: **Timelike bi-quadratic shifted-knots Bézier surface**

The timelike bi-quadratic SKBS in Minkowski space-$E_{1}^{3}$ can be obtained from the timelike SKBS Eq (2.6), ***ω***(*u*, *v*), (for *m* = 2 and *n* = 2),

$$\begin{array}{c} \omega \left(u,v\right)=\sum_{k,l=0}^{2,2}S_{♭,\varsigma }^{k,2}(u)S_{♭,\varsigma }^{l,2}(v)P_{kl}, \end{array}$$ (4.1)

and for $♭=0.2,\varsigma =0.8,P_{kl}^{\left(1,0\right)}$ and $P_{kl}^{\left(0,1\right)}$, the weighted forward differences of control points with *k*, *l* = 0, 1 can be determined using Eqs (2.23) and (2.25) at the minimum point (*u*, *v*) = (0, 0), which are

$$\begin{array}{c} \begin{array}{cc} & P_{00}^{\left(1,0\right)}=(3,6,14),\;P_{01}^{\left(1,0\right)}=(3,6,6),\;P_{10}^{\left(1,0\right)}=(3,0,-6),\\ & P_{00}^{\left(0,1\right)}=(0,8,8),\;P_{01}^{\left(0,1\right)}=(0,6,3),\;P_{10}^{\left(0,1\right)}=(0,8,0). \end{array} \end{array}$$ (4.2)

The fundamental coefficients *E*, *F* and *G* defined in Eq (3.5) can be determined for the control points specified in above Eq (4.2). The result shows that *E*, *F* and *G* are

$$\begin{array}{c} E=-151,\;F=-64,\;G=0. \end{array}$$ (4.3)

By utilizing the values of *E*, *F* and *G* found in the above Eq (4.3), the Lorentzian-metric of the surface ***ω***(*u*, *v*) given in Eq (3.7) can be expressed in the form,

$$\begin{array}{c} ds^{2}=-151du^{2}-64dudv. \end{array}$$ (4.4)

The second-order derivatives ***ω***_*uu*_(0, 0), ***ω***_*uv*_(0, 0) and ***ω***_*vv*_(0, 0) of the SKBS can be calculated by using the control points determined in Eq (4.2), as described in Eqs (2.34) to (2.36), and they turn out to be

$$\begin{array}{c} \omega_{uu}\left(0,0\right)=(0,-8,-27),\;\omega_{uv}\left(0,0\right)=(0,0,-24),\;\omega_{vv}\left(0,0\right)=(0,-4,-8). \end{array}$$ (4.5)

The unit normal vector $N\left(0,0\right)=\frac{P_{00}^{\left(1,0\right)}\wedge_{L}\;P_{00}^{\left(0,1\right)}}{\left\|P_{00}^{\left(1,0\right)}\wedge_{L}P^{\left(0,1\right)}\right\|}$ to SKBS, as defined in Eq (3.15), can be obtained by using the control points provided in Eq (4.2) at the point (*u*, *v*) = (0, 0), resulting in,

$$\begin{array}{c} N\left(0,0\right)=(1,\frac{3}{8},\frac{3}{8}), \end{array}$$ (4.6)

and hence the norm of the unit normal **N** in this case,

$$\begin{array}{c} \xi_{L}(N(0,0),N(0,0))=1>0, \end{array}$$ (4.7)

indicates that it is a spacelike-vector and the Eqs (4.5) and (4.6) enable us to find the coefficients (*e*, *f*, *g*) and they are

$$\begin{array}{c} e=\frac{57}{8},\;f=9,\;g=\frac{3}{2}, \end{array}$$ (4.8)

and from Eq (4.3), we can find det(*η*) = *EG* − *F*^2^ and it is given by,

$$\begin{array}{c} \text{det}(\eta )=-{\left(64\right)}^{2}<0. \end{array}$$ (4.9)

Now, from Eqs (4.3) and (4.9), the matrix-coefficients λ_11_, λ_12_, λ_21_ and λ_22_ of the matrix λ turn out to be,

$$\begin{array}{c} \lambda_{11}=-\frac{9}{64},\;\lambda_{12}=-\frac{3}{128},\;\lambda_{21}=\frac{903}{4096},\;\lambda_{22}=-\frac{699}{8192}. \end{array}$$ (4.10)

For *ϵ* = 1, Gaussian and mean-curvature as given in Eq (2.19) can now be computed by using the matrix-coefficients λ_11_, λ_12_, λ_21_ and λ_22_ as given in the above Eq (4.10), and they are found to be,

$$\begin{array}{c} K=\frac{1125}{65536},\;H=\frac{1851}{16384},\;. \end{array}$$ (4.11)

**Fig 2:**
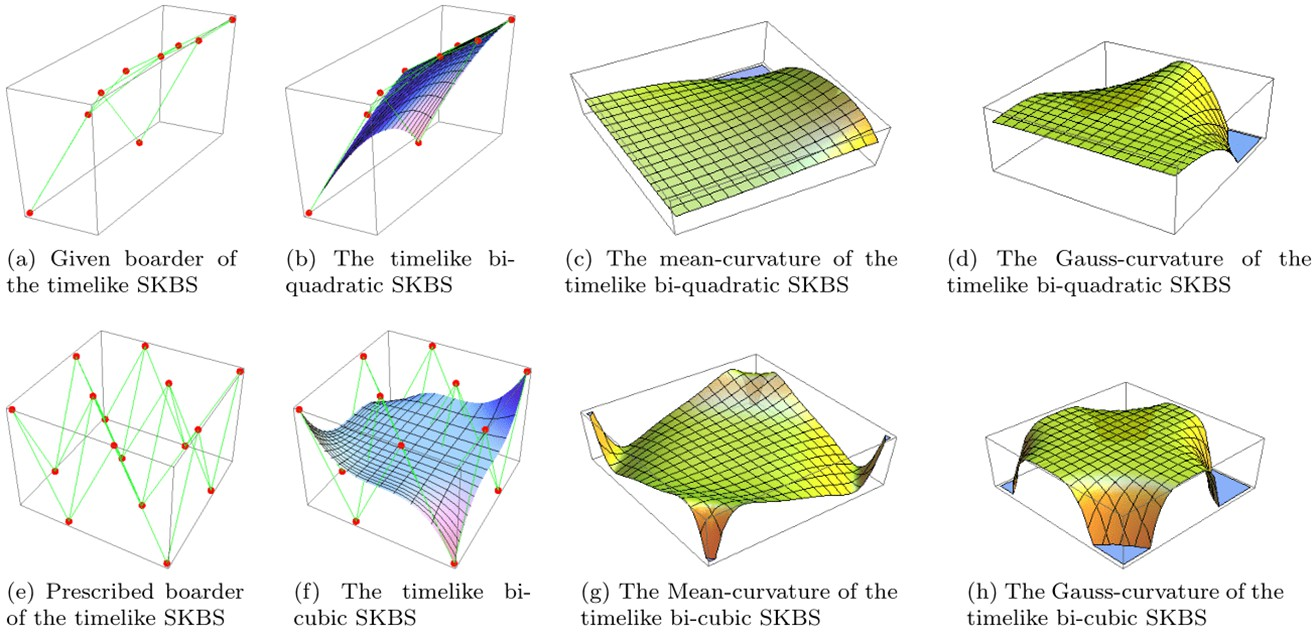
Timelike shifted-knots Bézier surfaces. The timelike bi-quadratic and bi-cubic SKBS at ♭=0.2 and ς=0.8.

Fig 2(a)–2(d) display the boarder and the timelike bi-quadratic SKBS along with its mean curvature and Gaussian curvature functions for shape parameters ♭=0.2 and ς=0.8, respectively.

**Case-2**: **Timelike bi-cubic shifted-knots Bézier surface**

The timelike bi-cubic shifted-knots Bézier surface (for *m* = 3 and *n* = 3) in Minkowski space-$E_{1}^{3}$ can be written from the timelike SKBS (Eq (2.6)) for (*u*, *v*) ∈ [0, 1] × [0, 1],

$$\begin{array}{c} \omega \left(u,v\right)=\sum_{k,l=0}^{3,3}S_{♭,\varsigma }^{k,3}(u)S_{♭,\varsigma }^{k,3}(v)P_{kl}. \end{array}$$ (4.12)

The weighted forward differences of control points, $P_{kl}^{\left(1,0\right)}$ and $P_{kl}^{\left(0,1\right)}$ can be determined from the Eqs (2.23) and (2.25) for *k*, *l* = 0, 1 at the minimum point (*u*, *v*) = (0, 0), which are

$$\begin{array}{c} \begin{array}{cc} & P_{00}^{\left(1,0\right)}=(4,0,-8),\;P_{01}^{\left(1,0\right)}=(4,0,8),\;P_{10}^{\left(1,0\right)}=(4,0,8),\\ & P_{00}^{\left(0,1\right)}=(0,4,-8),\;P_{01}^{\left(0,1\right)}=(0,4,8),\;P_{10}^{\left(0,1\right)}=(0,4,8). \end{array} \end{array}$$ (4.13)

By plugging the Eq (4.13) into Eq (3.5) (in the corollary (3.1.1)), we can compute the coefficients *E*, *F*, and *G* of the first-fundamental form of the timelike bi-cubic SKBS in Minkowski space-$E_{1}^{3}$. The resulting values are

$$\begin{array}{c} E=-48,\;F=-64,\;G=-48. \end{array}$$ (4.14)

This leads to the corresponding Lorentzian-metric (3.7) of the timelike bi-cubic SKBS, which is given by

$$\begin{array}{c} ds^{2}=-48du^{2}-64dudv-48dv^{2}. \end{array}$$ (4.15)

The second-order derivatives of the timelike bi-cubic SKBS in Minkowski space-$E_{1}^{3}$ are obtained from the Corollary (2.3.1) as well,

$$\begin{array}{c} \omega_{uu}\left(0,0\right)=(0,0,39),\;\omega_{uv}\left(0,0\right)=(0,0,58),\;\omega_{vv}\left(0,0\right)=(0,0,39). \end{array}$$ (4.16)

This enables us to determine the unit normal-vector **N** of the timelike bi-cubic SKBS in Minkowski space-$E_{1}^{3}$ by utilizing Eq (3.15) from the Corollary (3.4.1) and Eq (4.13), given by

$$\begin{array}{c} N\left(0,0\right)=\frac{1}{\sqrt{7}}(2,2,1), \end{array}$$ (4.17)

and in this case, it appears that the normal **N**(*u*, *v*) is spacelike-vector as the norm of the normal vector **N**(*u*, *v*) is

$$\begin{array}{c} \xi_{L}(N(0,0),N(0,0))=1>0. \end{array}$$ (4.18)

The fundamental coefficients *e*, *f* and *g* of the of the timelike bi-cubic SKBS can be obtained from Eqs (4.16) and (4.17) to obtain,

$$\begin{array}{c} e=-\frac{39}{\sqrt{7}},\;f=-\frac{58}{\sqrt{7}},\;g=-\frac{39}{\sqrt{7}}. \end{array}$$ (4.19)

By the fundamental coefficients obtained in the Eq (4.14), the determinant det(*η*) is

$$\begin{array}{c} \text{det}(\eta )=EG-F^{2}=-1792<0. \end{array}$$ (4.20)

The coefficients λ_11_, λ_12_, λ_21_, and λ_22_ for the matrix λ of the shape-operator of the timelike bi-cubic SKBS can be obtained through the use of Eqs (4.14), (4.19) and (4.20)

$$\begin{array}{c} \lambda_{11}=\frac{115}{112\sqrt{7}},\;\lambda_{12}=\frac{-9}{56\sqrt{7}},\;\lambda_{21}=\frac{-9}{56\sqrt{7}},\;\lambda_{22}=\frac{115}{112\sqrt{7}}. \end{array}$$ (4.21)

Utilizing the shape-operator defined in Eq (4.21) for the timelike bi-cubic SKBS for *ϵ* = 1, the Gauss-curvature and the mean-curvature defined by Eq (2.19) are found to be

$$\begin{array}{c} K=\frac{1843}{12544},\;H=\frac{115}{112\sqrt{7}}. \end{array}$$ (4.22)

Fig 2(e)–2(h) represent the boarder and the timelike bi-cubic SKBS along with its mean curvature and Gaussian curvature for shape parameters ♭=0.2 and ς=0.8, respectively.

**Case-3**: **Spacelike bi-quadratic shifted-knots Bézier surface**

The weighted forward differences of control points $P_{kl}^{\left(1,0\right)}$ and $P_{kl}^{\left(0,1\right)}$, can be found using the Eqs (2.23) and (2.25) for *k*, *l* = 0, 1 at the minimum point (*u*, *v*) = (0, 0), which are

$$\begin{array}{c} \begin{array}{cc} & P_{00}^{\left(1,0\right)}=(3,0,0),\;P_{01}^{\left(1,0\right)}=(3,0,3),\;P_{10}^{\left(1,0\right)}=(3,0,0),\\ & P_{00}^{\left(0,1\right)}=(0,3,0),\;P_{01}^{\left(0,1\right)}=(0,3,0),\;P_{10}^{\left(0,1\right)}=(0,3,3). \end{array} \end{array}$$ (4.23)

The fundamental coefficients *E*, *F* and *G* of the spacelike bi-quadratic SKBS in the Minkowski space-$E_{1}^{3}$ can be obtained from the Eq (4.23), resulting in,

$$\begin{array}{c} E=9,\;F=0,\;G=9. \end{array}$$ (4.24)

The Lorentzian-metric (3.7) of the spacelike bi-quadratic SKBS in this case is given by,

$$\begin{array}{c} ds^{2}=9du^{2}+9dv^{2}. \end{array}$$ (4.25)

The second-order derivatives ***ω***_*uu*_(0, 0), ***ω***_*uv*_(0, 0) and ***ω***_*vv*_(0, 0) of the spacelike bi-quadratic SKBS in Minkowski space-$E_{1}^{3}$ can be obtained from the Eqs (2.34) to (2.36) and they turnout to be

$$\begin{array}{c} \omega_{uu}\left(0,0\right)=(0,0,0),\;\omega_{uv}\left(0,0\right)=(0,0,8),\;\omega_{vv}\left(0,0\right)=(0,0,0). \end{array}$$ (4.26)

By inserting the values of the weighted forward differences of control points $P_{kl}^{\left(1,0\right)}$ and $P_{kl}^{\left(0,1\right)}$, obtained from Eq (4.23) for *k*, *l* = 0, 1 into the Eq (3.15), we can find the unit normal **N** to spacelike bi-quadratic SKBS in Minkowski space-$E_{1}^{3}$, yielding,

$$\begin{array}{c} N\left(0,0\right)=(0,0,1). \end{array}$$ (4.27)

For the Minkowski-metric, the norm of the unit normal (4.27) turns out to be,

$$\begin{array}{c} \varphi (N(0,0),N(0,0))=-1<0, \end{array}$$ (4.28)

which means that the normal vector **N** is a timelike-vector. The fundamental coefficients *e*, *f* and *g* of the spacelike bi-quadratic SKBS, can be determined using Eqs (4.26) and (4.27)) and they are

$$\begin{array}{c} \begin{array}{cc} & e=0,\;f=-8,\;g=0, \end{array} \end{array}$$ (4.29)

for a timelike surface, *ϵ* = −1. Now, from the fundamental coefficients given in the Eq (4.24), it can be seen that

$$\begin{array}{c} \text{det}(\eta )=EG-F^{2}=81>0. \end{array}$$ (4.30)

The coefficients λ_11_, λ_12_, λ_21_, and λ_22_ of the matrix λ corresponding to the shape-operator of the spacelike bi-quadratic SKBS, can be obtained from the Eqs (4.24) and (4.30), and they are

$$\begin{array}{c} \begin{array}{cc} & \lambda_{11}=0,\;\lambda_{12}=-\frac{8}{9},\;\lambda_{21}=-\frac{8}{9},\;\lambda_{22}=0. \end{array} \end{array}$$ (4.31)

Gaussian and mean-curvature can be calculated by using the matrix coefficients λ_11_, λ_12_, λ_21_ and λ_22_ as described in Eq (4.31), for a value of *ϵ* equal to -1, by utilizing Eq (2.19),

$$\begin{array}{c} \begin{array}{cc} & K=\frac{64}{81},\;H=0. \end{array} \end{array}$$ (4.32)

**Fig 3:**
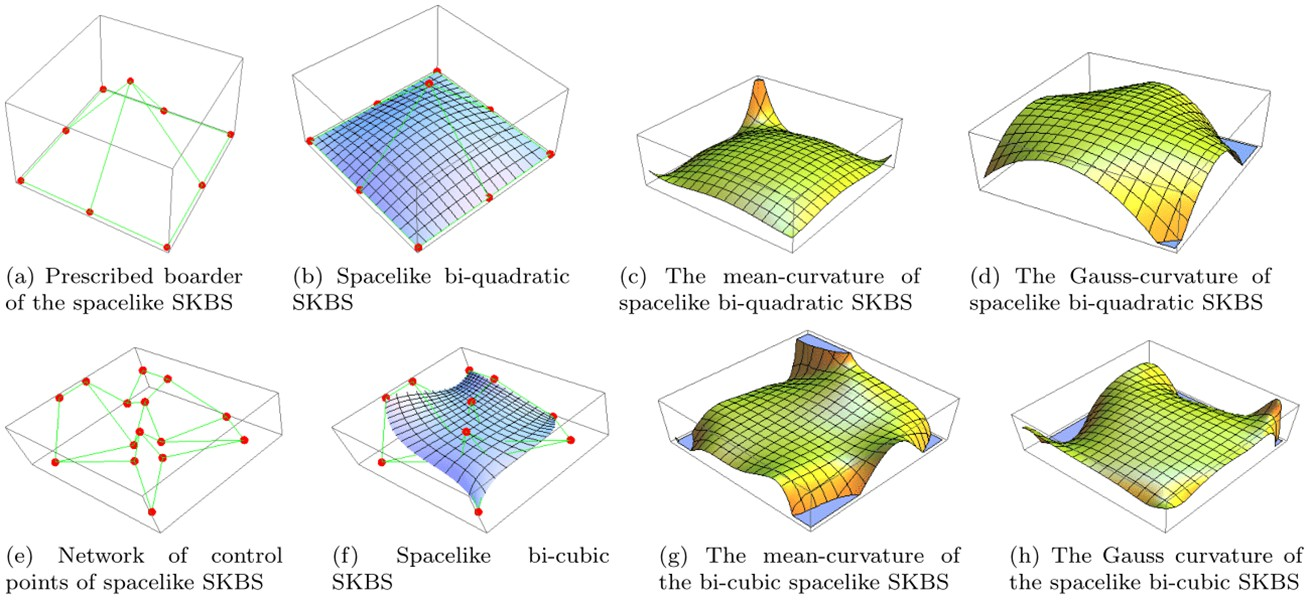
Spacelike shifted-knots Bézier surfaces. The spacelike bi-quadratic and bi-cubic shifted-knots Bézier surface ♭=0.2 and ς=0.8.

Fig 3 presents the shape-operator quantities, such as the boundary control points, the bi-quadratic surface itself, and the mean and Gaussian curvature function of the spacelike bi-quadratic SKBS.

**Case-4**: **Spacelike bi-cubic shifted-knots Bézier surface**

The spacelike bi-cubic SKBS in Minkowski space-$E_{1}^{3}$ is given in Eq (4.12). The Lorentzian metric (3.7) of the surface ***ω***(*u*, *v*) at the point (0, 0) can be determined by using Eq (2.23) and the Eq (2.25) for the weighted forward differences and they turn out to be

$$\begin{array}{c} \begin{array}{cc} & P_{00}^{\left(1,0\right)}=(6,5,0),\;P_{01}^{\left(1,0\right)}=(2,0,4),\;P_{10}^{\left(1,0\right)}=(6,-5,0),\\ & P_{00}^{\left(0,1\right)}=(5,6,0),\;P_{01}^{\left(0,1\right)}=(-5,6,0),\;P_{10}^{\left(0,1\right)}=(0,2,4). \end{array} \end{array}$$ (4.33)

The coefficients *E*, *F* and *G* of the first-fundamental form of the spacelike bi-cubic SKBS in the Minkowski space-$E_{1}^{3}$ can be calculated from the Eq (4.33) and they are

$$\begin{array}{c} E=61,\;F=60,\;G=61. \end{array}$$ (4.34)

Utilizing the fundamental coefficients (4.34), the corresponding Lorentzian-metric (3.7) for spacelike bicubic surface in this case is given by

$$\begin{array}{c} ds^{2}=61du^{2}+60dudv+61dv^{2}. \end{array}$$ (4.35)

For the spacelike bi-cubic SKBS, ***ω***(*u*, *v*) in Minkowski space-$E_{1}^{3}$, the second order derivatives, ***ω***_*uu*_(0, 0), ***ω***_*uv*_(0, 0) and ***ω***_*vv*_(0, 0) can be obtained from the Eqs (2.34) to (2.36) and they are

$$\begin{array}{c} \omega_{uu}\left(0,0\right)=(0,-23,0),\;\omega_{uv}\left(0,0\right)=(-17,-17,14),\;\omega_{vv}\left(0,0\right)=(-23,0,0). \end{array}$$ (4.36)

By using the weighted forward differences of control points $P_{kl}^{\left(1,0\right)}$ and $P_{kl}^{\left(0,1\right)}$ from Eq (4.33) for *k*, *l* = 0, 1 and inserting these values into Eq (3.15), the unit normal **N** to the spacelike bi-cubic SKBS in Minkowski space-$E_{1}^{3}$ can be found and it is given by,

$$\begin{array}{c} N\left(0,0\right)=(0,0,1). \end{array}$$ (4.37)

The norm of the unit normal (4.37) turns out to be

$$\begin{array}{c} \varphi (N(0,0),N(0,0))=-1<0, \end{array}$$ (4.38)

and hence in this case, the unit normal N(u,v) is a timelike-vector. The fundamental coefficients *e*, *f* and *g* of the spacelike bi-cubic SKBS can be determined from the Eqs (4.36) and (4.37)). It turns out that,

$$\begin{array}{c} \begin{array}{cc} & e=0,\;f=-14,\;g=0, \end{array} \end{array}$$ (4.39)

as for a timelike surface, *ϵ* = −1. In this case, for the fundamental coefficients given in the Eq (4.24), note that

$$\begin{array}{c} \text{det}(\eta )=121>0. \end{array}$$ (4.40)

By using the Eqs (4.34) and (4.40), the matrix coefficients of the shape operator of the spacelike bi-cubic SKBS, λ_11_, λ_12_, λ_21_, and λ_22_, are determined as follows,

$$\begin{array}{c} \begin{array}{cc} & \lambda_{11}=\frac{840}{121},\;\lambda_{12}=-\frac{854}{121},\;\lambda_{21}=-\frac{854}{121},\;\lambda_{22}=\frac{840}{121}. \end{array} \end{array}$$ (4.41)

By using the shape-operator defined in the Eq (4.41), the Gauss-curvature and mean-curvature of the spacelike bi-cubic shifted-knots Bézier surface can be found for *ϵ* = −1 and it follows that

$$\begin{array}{c} \begin{array}{cc} & K=\frac{196}{121},\;H=\frac{840}{121}. \end{array} \end{array}$$ (4.42)

Fig 3 shows a network of given control points for the spacelike bi-cubic shifted-knots Bézier surface, the SKBS itself, its mean and Gaussian curvature for ♭=0.2 and ς=0.8.

## 5 Conclusion

This study explores the properties of timelike and spacelike shifted-knots Bézier surface in Minkowski space-$E_{1}^{3}$. By computing the fundamental coefficients of these surfaces, including the Gauss curvature, mean curvature, and shape operator, this study provides a deeper understanding of these surfaces from a geometric perspective and their potential applications in the fields of CAGD and CG. The numerical examples presented in this study demonstrate the feasibility of the methodology and offer further insight into the characteristics of timelike and spacelike shifted-knots Bézier surface in Minkowski space. The results of this study open avenues for future research, including the investigation of other surfaces such as *q*-Bernstein Bézier surfaces, (*p*, *q*) Bernstein Bézier surfaces, quasi-cubic Bézier surfaces, and quasi-quintic Bézier surfaces. These surfaces can be utilized in the optimization of energy functionals to obtain minimal, quasi-harmonic, and bi-harmonic surfaces as their extremals. The results of this study suggest avenues for further research, such as exploring other surfaces and optimizing energy functionals. These findings have the potential to advance geometric modeling and visualization techniques.
